# Rice Dwarf Virus P2 Protein Hijacks Auxin Signaling by Directly Targeting the Rice OsIAA10 Protein, Enhancing Viral Infection and Disease Development

**DOI:** 10.1371/journal.ppat.1005847

**Published:** 2016-09-08

**Authors:** Lian Jin, Qingqing Qin, Yu Wang, Yingying Pu, Lifang Liu, Xing Wen, Shaoyi Ji, Jianguo Wu, Chunhong Wei, Biao Ding, Yi Li

**Affiliations:** 1 The State Key Laboratory of Protein and Plant Gene Research, College of Life Sciences, Peking University, Beijing, People’s Republic of China; 2 Department of Molecular Genetics and Center for RNA Biology, The Ohio State University, Columbus, Ohio, United States of America; University of California, Davis Genome Center, UNITED STATES

## Abstract

The phytohormone auxin plays critical roles in regulating myriads of plant growth and developmental processes. Microbe infection can disturb auxin signaling resulting in defects in these processes, but the underlying mechanisms are poorly understood. Auxin signaling begins with perception of auxin by a transient co-receptor complex consisting of an F-box transport inhibitor response 1/auxin signaling F-box (TIR1/AFB) protein and an auxin/indole-3-acetic acid (Aux/IAA) protein. Auxin binding to the co-receptor triggers ubiquitination and 26S proteasome degradation of the Aux/IAA proteins, leading to subsequent events, including expression of auxin-responsive genes. Here we report that *Rice dwarf virus* (RDV), a devastating pathogen of rice, causes disease symptoms including dwarfing, increased tiller number and short crown roots in infected rice as a result of reduced sensitivity to auxin signaling. The RDV capsid protein P2 binds OsIAA10, blocking the interaction between OsIAA10 and OsTIR1 and inhibiting 26S proteasome-mediated OsIAA10 degradation. Transgenic rice plants overexpressing wild-type or a dominant-negative (degradation-resistant) mutant of OsIAA10 phenocopy RDV symptoms are more susceptible to RDV infection; however, knockdown of *OsIAA10* enhances the resistance of rice to RDV infection. Our findings reveal a previously unknown mechanism of viral protein reprogramming of a key step in auxin signaling initiation that enhances viral infection and pathogenesis.

## Author Summary

Auxin regulates plant growth and development through auxin signaling, which begins with the interaction of an F-box transport inhibitor response 1/auxin signaling F-box (TIR1/AFB) protein and an auxin/indole-3-acetic acid (Aux/IAA) protein co-receptor. Auxin binding to the co-receptor complex triggers ubiquitination and 26S proteasome degradation of Aux/IAA proteins, leading to a downstream signaling cascade that induces the expression of auxin-responsive genes. Auxin signaling is manipulated by plant pathogens to maximize their own multiplication, but the underlying mechanisms are poorly understood. Here we report that the P2 capsid protein encoded by *Rice dwarf virus* (RDV) sabotages auxin signaling by interacting with the rice Aux/IAA protein, OsIAA10, thereby shielding it from degradation and causing infected plants to display typical RDV symptoms including dwarfism, excessive tillering and stunted crown roots. Importantly, these symptoms are phenocopied by transgenic rice plants overexpressing OsIAA10 or its degradation-resistant mutant. Conversely, down-regulating OsIAA10 expression in rice led to milder RDV infection. Together these findings reveal a novel mechanism by which RDV reprograms auxin signaling, leading to enhanced viral infection.

## Introduction

Viral infection causes enormous losses in crop yield and crop quality, posing a constant threat to food security. These losses are attributed to virus-induced abnormal growth and development, exhibited as disease symptoms. However, The molecular basis of disease symptom development in plants remains poorly understood [1–5]. Plant hormones control many aspects of the plant growth and development by orchestrating the expression of plant genes in a temporally and spatially regulated manner, and by coordinating plant responses to environmental cues. Perturbation of hormone signaling in plant often causes developmental defects, some of which share characteristics with virus-induced disease symptoms [2,6]. Viral infections also interfere with plant hormone homeostasis [6–10].

Auxin is one of the major phytohormones that controls many aspects of plant growth and development [11,12]. Auxin signaling begins with perception of auxin by a transient co-receptor complexes consisting of an auxin/indole-3-acetic acid (Aux/IAA) protein and an F-box transport inhibitor response1/auxin signaling F-box (TIR1/AFB) protein [13–18]. Auxin binding to the co-receptor triggers the ubiquitination of Aux/IAA proteins by TIR1/AFBs and their degradation by the 26S proteasome, causing derepression of auxin response factors (ARFs), a class of transcriptional regulators that are sequestered by Aux/IAAs through heterodimerization or oligomerization. Then the ARFs reprogram the transcription of auxin-responsive genes, resulting in adjustments to plant growth and/or development [12,18,19]. Genes that are rapidly and transiently induced or repressed by auxin include Small Auxin-up RNAs (SAURs), GH3 proteins, and Aux/IAA proteins [11,20].

Aux/IAA proteins are at the nexus of the auxin signaling. A total of 31 and 29 *Aux/IAA* genes have been found in rice [21] and Arabidopsis [22] genomes, respectively, most of which encode proteins containing four conserved domains designated domains I, II, III, and IV. Domain I confers repressor activity by recruiting the Topless1 corepressor, whereas domain II is responsible for interaction with TIR1. Domains III and IV, later referred to as the Phox/Bem 1 (PB1) domain, mediates interactions with ARFs as well as other Aux/IAA proteins [20,22–30]. Mutations in domain II have been shown to disrupt the interaction of Aux/IAAs with TIR1, causing stabilization of Aux/IAA proteins [24]. The resulting mutant plants are insensitive to auxin treatment, and display characteristic Aux/IAA gain-of-function phenotypes including stunted stature, decreased apical dominance, low fertility, and darker mature leaves [31–36].

Aux/IAA gain-of-function mutant plants share striking phenotypic resemblance with rice plants infected by the *Rice dwarf virus* (RDV). RDV, a member of the genus *Phytoreovirus* in *Reoviridae* family, is transmitted by the green rice leafhopper (*Nephotettix cincticeps*), and is a devastating pathogen that periodically causes rice yield losses. Its genome comprises 12-segmented double-stranded RNAs (dsRNAs) that encode seven structural proteins (P1, P2, P3, P5, P7, P8, and P9) [10] and at least five nonstructural proteins (Pns4, Pns6, Pns10, Pns11, and Pns12) [37–39]. RDV-infected plants exhibit disease symptoms including severe stunted growth, increased tiller number, and shorter and fewer roots [10,40], which resemble the phenotypes of Aux/IAA gain-of-function mutant plants described above [31,34–36]. The morphological similarities shared by RDV-infected rice plants and mutant plants defective in auxin signaling suggest that RDV infection might manipulate auxin signaling cascades.

In this study, we demonstrate that RDV infection interferes with auxin signaling in rice plants by stabilizing the rice Aux/IAA protein, OsIAA10. This occurs through a highly specific interaction between the RDV P2 protein and domain II of OsIAA10, which thwarts the interaction of OsIAA10 with OsTIR1, thereby preventing its degradation upon auxin perception. In addition, we show that this interaction causes alterations in rice morphogenesis and is needed for optimal RDV infection. Finally, we show that knock-down of OsIAA10 enhances the resistance of rice to RDV infection, whereas OsIAA10 overexpression has the opposite effect. Together, these data show the novel mechanism used by which RDV reprograms auxin signaling cascades, resulting in enhanced viral infection and disease development.

## Results

### RDV infection alters the response of rice to auxin

**Fig 1:**
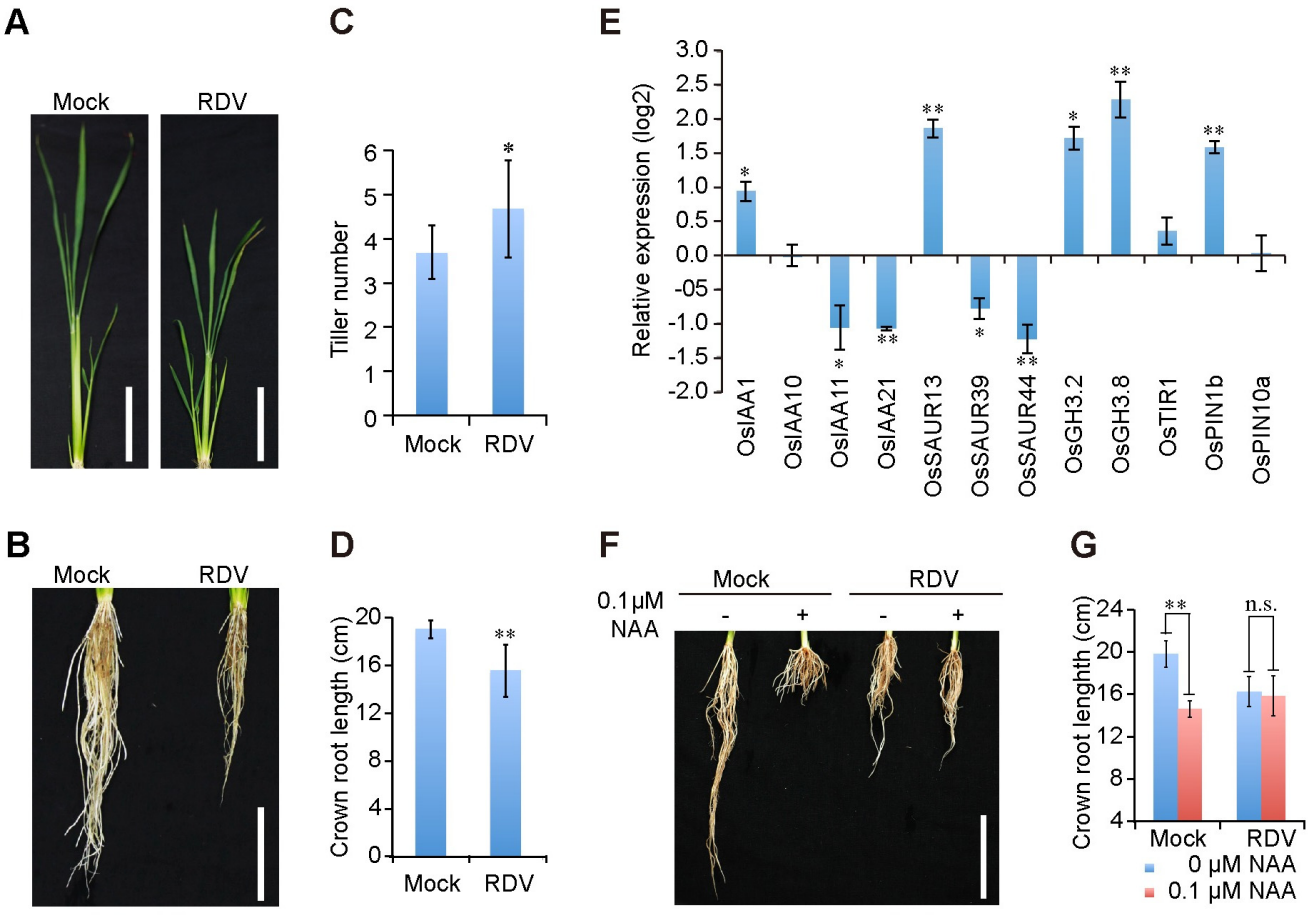
RDV infection disturbs auxin pathway in rice. (A and B) Aboveground (A) and root (B) phenotypes of mock- and RDV- infected rice plants at 6-week-old seedling stage. Bars: 10 cm. (C and D) Schematic representation of the tiller number and crown roots length of mock- and RDV- infected rice plants in (A) and (B). The average (± standard deviation (SD)) values were obtained from three biological repeats, with 15 plants from each line in every repeat. Significant differences were indicated (*P<0.05, **P<0.01) based on Student’s *t*-test. (E) Relative average expression (log2) of auxin-induced genes in RDV-infected rice plants. Data were obtained from qPCR assays and analyzed using 2^-ΔΔC(t)^ method and the *OsEF1a* mRNA levels were used as internal controls. Values are mean ± SD (n = 3 biological replicates). Columns with asterisks are statistically different according to Student’s *t*-test (*P<0.05, **P<0.01) as compared to their expression in mock-inoculated rice plants. (F and G) RDV-infected rice plants exhibit reduced sensitivity to auxin treatment. Phenotypes (F) and lengths (G) of crown roots of mock- and RDV- infected 4-week-old seedlings cultured in liquid nutrition containing 0 or 0.1 μM NAA for 10 days. Bar: 10 cm. The average (± SD) values were from three biological repeats with 15 plants for each line every repeat. Significant differences were indicated (n.s., no significant, **P<0.01) based on Student’s *t*-test.

We were interested in determining whether RDV infection interferes with auxin signaling in rice plants because symptoms in RDV-infected plants, including severe stunting, increased tiller number, and shorter crown roots (Fig 1A–1D), are strikingly similar to the auxin-resistant phenotypes caused by transgenic overexpression of microRNA393 (miR393), which targets mRNAs encoding *TIR1/AFB2* [16,41–43]), or OsIAA4 [43,44]. To assess whether the auxin signaling pathway is indeed compromised by RDV infection, we used quantitative real-time PCR (qPCR) to analyze changes in the expression levels of a panel of auxin-related genes upon RDV infection. The results showed that RDV infection caused significant changes in the expression of *OsIAA21*, *OsSAUR13*, *OsSAUR44*, *OsGH3*.*8* and *OsPIN1b*, and less pronounced changes in the expressions of *OsIAA1*, *OsIAA11*, *OsSAUR39*, and *OsGH3*.*2* (Fig 1E). This pattern of extensive perturbation of auxin signaling genes at the transcription level is consistent with the previously reported microarray analyses [45], suggesting that RDV manipulates auxin signaling pathways.

To further test the auxin responsiveness of RDV-infected rice plants, we subjected them to treatment with the auxin analogue α-naphthalene acetic acid (NAA). As shown in Fig 1F and 1G, elongation of the crown roots of mock-inoculated rice seedlings was significantly inhibited by 0.1μM NAA; however RDV-infected rice seedlings with or without NAA treatment grew much shorter crown roots compared to control seedling (Fig 1F and 1G). There was no significant difference between treated and untreated RDV-infected rice (Fig 1F and 1G). These data show that RDV infection causes rice plants to be less responsive to auxin application.

### The RDV P2 protein interacts with OsIAA10

**Fig 2:**
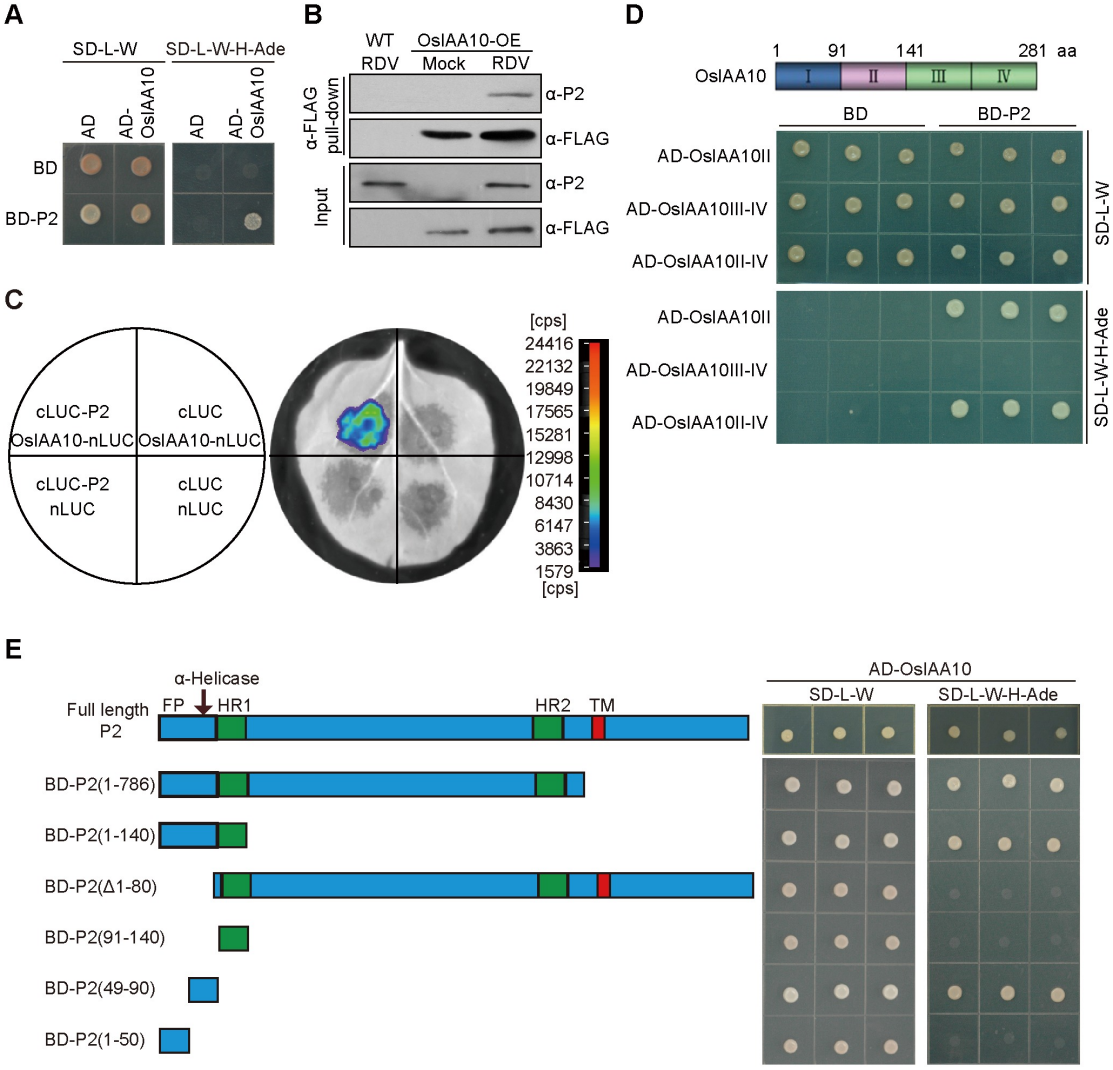
The RDV P2 protein interacts with OsIAA10. (A) RDV P2 interacts with OsIAA10 in yeast. Yeast transformants were spotted on the control medium (SD-Leu/-Trp (SD-L-W)) and selection medium (SD-Leu/-Trp/-His/-Ade (SD-L-W-H-Ade)). AD, activating domain; BD, binding domain; SD, synthetic dropout. (B) Co-immunoprecipitation confirms the interaction between P2 and OsIAA10 in RDV-infected FLAG-OsIAA10 overexpressing (OsIAA10-OE) rice. WT, wild type rice; Mock, mock-inoculated rice; RDV, RDV-infected rice. (C) LCI assay shows interaction between P2 and OsIAA10 *in vivo*. The left diagram indicates the leaf panels that were infiltrated with *A*. *tumefaciens* containing the different combinations of indicated constructs. Cps indicates signal counts per second. (D) P2 interacts with domain II of OsIAA10. (E) Determination of the functional domains of P2 that interact with OsIAA10. The prey protein AD-OsIAA10 was expressed with the indicated bait proteins in yeast AH109 cells. Interaction was indicated by the ability of cells to grow on medium SD-L-W-H-Ade.

Next, we determined the mechanism by which RDV infection perturbs auxin signaling. The P2 protein encoded by RDV is a multi-functional protein that, in addition to being part of the viral capsid, induces dwarfism in RDV-infected rice by binding to *ent*-kaurene oxidases, thereby disrupting gibberellins biosynthesis [10, 46]. To assess its potential interference with auxin signaling, we used the yeast two-hybrid screening assay to identify candidate rice proteins that physically interact with RDV P2. Our initial screening revealed a rice cDNA encoding the C-terminal residues 91–280 of OsIAA10. The interaction between P2 and full-length OsIAA10 in yeast was also confirmed in this assay (Fig 2A). The *in planta* interaction was verified by co-immunoprecipitation (co-IP) of endogenous P2 with overexpressed FLAG-OsIAA10 in RDV-infected transgenic rice plants (Fig 2B), and by co-IP of transiently overexpressed FLAG-OsIAA10 and HA-P2 in *Nicotiana benthamiana* (*N*. *benthamiana*) leaves (S1A Fig). A firefly luciferase complementation imaging (LCI) assay also showed the interaction of the two proteins (Fig 2C). OsIAA10 belongs to the Aux/IAA protein family, of which 31 members have been identified in rice (S1B Fig) [21]. The RDV P2 protein specifically interacts with OsIAA10, but not with the 19 other OsIAAs that have varying degrees of relatedness to OsIAA10 (S1C Fig). Conversely, among all of the RDV-encoded proteins, P2 is the only one that interacts with OsIAA10 (S1D Fig), indicating a high level of specificity. Similar to other Aux/IAA proteins, OsIAA10 contains four conserved domains (S2A Fig) [21,23], and is degraded through the ubiquitin-26S proteasome degradation pathway (S2B Fig) [19]. We also demonstrated the auxin-dependent interaction of OsIAA10 with the auxin receptor OsTIR1 (S2C Fig) through its domain II (S2D Fig). Finally, we mapped the P2-interacting of OsIAA10 to domain II (Fig 2D and S2D Fig).

### The RDV P2 protein stabilizes OsIAA10 by blocking its interaction with OsTIR1

Because both P2 and OsTIR1 bind OsIAA10 domain II (Fig 2D and S2D Fig) but P2 and OsTIR1 do not interact with each other (S3 Fig), we speculated that P2/OsIAA10 interaction might prevent OsTIR1 from accessing OsIAA10 by binding to the same interaction domain. To test this hypothesis, we performed an *in vitro* pull-down assay with purified recombinant proteins to assess whether P2 interferes with the OsIAA10/OsTIR1 interaction. Because the full-length P2 protein could not be adequately expressed in *Escherichia coli* (*E*. *coli*), we tried to map the minimal P2 region required for the OsIAA10 interaction using yeast two-hybrid assays. The results showed that P2 fragments P2(1–786), P2(1–140) and P2(49–90), all of which comprised amino acids (aa) 49–90, each interacted with OsIAA10, whereas fragments lacking these aa residues did not (Fig 2E).

**Fig 3:**
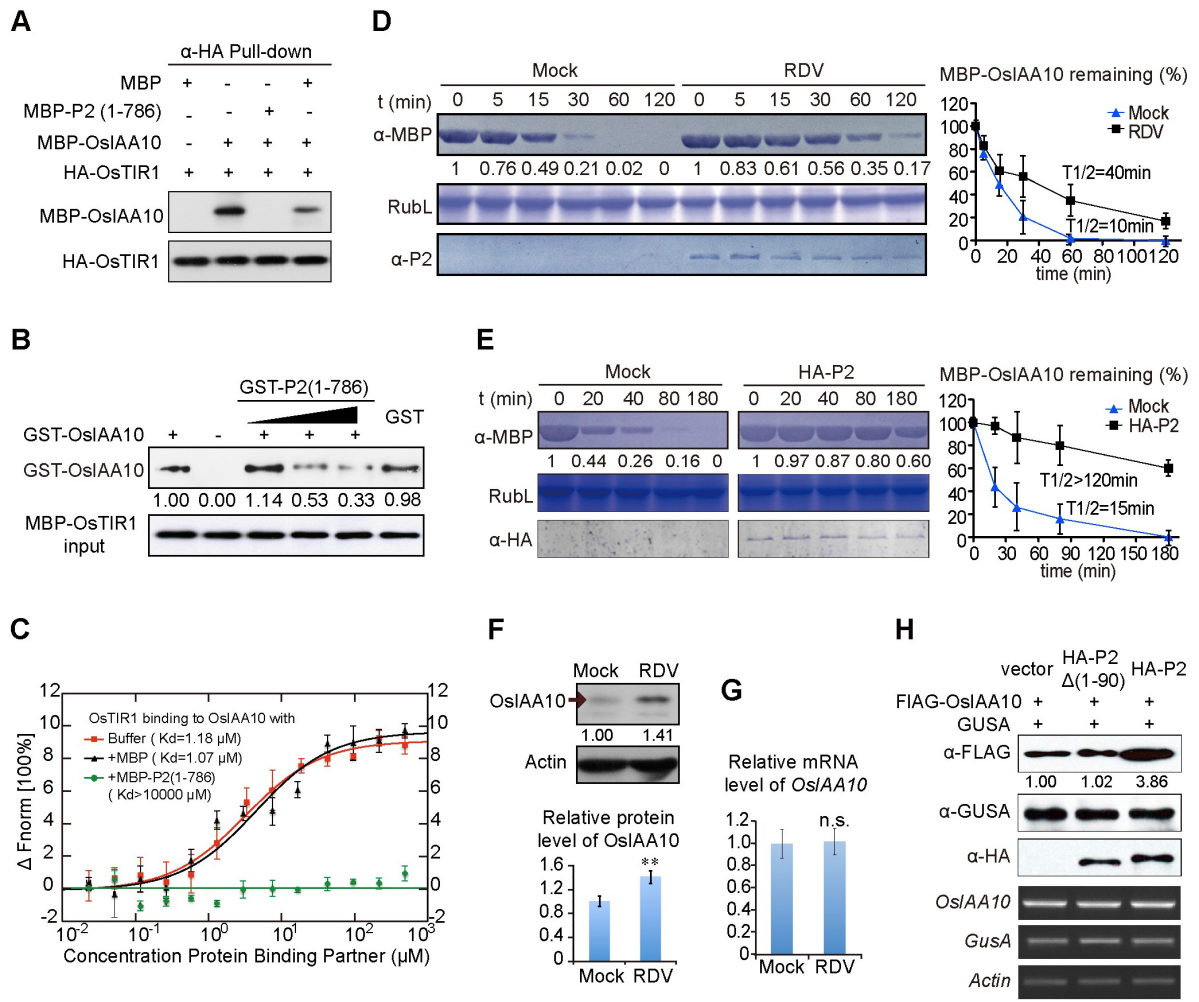
The RDV P2 protein stabilizes OsIAA10 by inhibiting OsIAA10/OsTIR1 interaction. (A) Interaction between MBP-OsIAA10 and HA-OsTIR1 is disrupted by MBP-P2 (1–786). MBP-OsIAA10 protein combined with MBP-P2(1–786) or MBP was incubated with immobilized HA-OsTIR1. The immunoprecipitated fractions were detected by anti-MBP antibody. HA-OsTIR1 input is shown in the lower panel. (B) *In vitro* interaction between GST-OsIAA10 and MBP-OsTIR1 is weakened by GST-P2 (1–786) in a dose dependent manner, revealed by pull-down. GST-OsIAA10 protein combined with GST-P2(1–786) or GST was incubated with immobilized MBP-OsTIR1. The immunoprecipitated fractions were detected by anti-GST antibody. The gradient indicates increasing amount of GST-P2(1–786). MBP-OsTIR1 input is shown in the lower panel. (C) P2 affects dynamic association between OsTIR1 and OsIAA10. Data were collected from microscale thermophoresis (MST) assays as described in Materials and Methods. Experiments repeat for three times and Error bars indicate SD. Fnorm, normalized fluorescence. (D and E) Cell-free degradation assay of MBP-OsIAA10 in mock- or RDV- infected rice extracts (D) or *N*. *benthamiana* leaf extracts (E). Mock in (D) indicates healthy rice extracts; Mock in (E), extracts of leaves infiltrated with *pWM101* vector as a negative control; HA-P2, extracts of leaves infiltrated with *pWM101-HAS2* that express HA-P2. Rubisco large protein (RuL) was used as a loading control of total plant protein. On the right was a normalized plot for the degradation of MBP-OsIAA10 of the left. The details for quantification and normalization are described in Materials and Methods. Error bars indicate SD. (F) Western blot showing OsIAA10 protein levels in mock and RDV-infected WT rice plants. Actin was used as a loading control. And the Histogram underneath represents the relative protein level. Experiments repeat for three times and Error bars indicate SD. Significant differences were indicated (**P<0.01) based on Student’s *t*-test. (G) qPCR showing *OsIAA10* transcript levels, respectively, in mock and RDV-infected WT rice plants. *OsEF1a* was used as the reference. Values are mean ± SD (n = 3 biological replicates). n.s. indicates no significant difference based on Student’s *t*-test. (H) Effects of P2 and P2Δ(1–90) on the accumulation of OsIAA10 in *N*. *benthamiana* after auxin treatment. The three upper panels show protein levels on Western blots and the three lower panels show mRNA levels revealed by RT-PCR. GusA was expressed and loaded as a reference control.

Next, We expressed P2 (1–786) in *E*. *coli* as a maltose binding protein (MBP)-P2 fusion protein, and purified it from the *E*. *coli* extracts. MBP alone as well as MBP-OsIAA10 were similarly purified from *E*. *coli*. HA-OsTIR1 was extracted from *N*. *benthamiana* leaves transiently expressing HA-OsTIR1 and immobilized onto anti-HA-affinity gel beads. The same amounts of MBP or MBP-OsIAA10, or MBP-OsIAA10 mixed with MBP-P2 (1–786) or MBP, were incubated with the immobilized HA-OsTIR1, respectively. The immunoprecipitated fractions were detected with the anti-MBP antibody. MBP-OsIAA10 was pulled down by HA-OsTIR1, and MBP-P2 (1–786) disrupted the interaction between MBP-OsIAA10 and HA-OsTIR1, whereas MBP did not (Fig 3A). Additionally, we performed dose dependent *in vitro* competitive pull-down assays with purified glutathione-S-transferase (GST)-tagged OsIAA10 and P2(1–786), and MBP-OsTIR1 proteins. As shown in Fig 3B, increasing amounts of GST-P2 (1–786) reduced the amount of GST-OsIAA10 bound to MBP-OsTIR1, indicating that P2 interferes with the OsIAA10/OsTIR1 interaction by binding to OsIAA10.

Then we used Microscale Thermophoresis (MST) [47,48] to quantify the binding affinity between OsIAA10 and OsTIR1, as well as the extent to which P2 interferes with the binding. In this experiment, OsTIR1 was labeled with the red fluorescent dye N-hydroxylsuccinimide (NHS), and the results showed that NHS-labeled OsTIR1 bound to OsIAA10 with a *K*_*d*_ value of 1.18 μM +/- 0.244 μM (Fig 3C). Addition of 2.5 μM free MBP did not result in significant changes in binding kinetics (*K*_*d*_ = 1.07 μM +/- 0.242 μM). In contrast, addition of 2.5 μM MBP-P2 (1–786) completely abolished OsTIR1/OsIAA10 binding (Fig 3C), further confirming that P2/OsIAA10 binding impedes the OsTIR1/OsIAA10 interaction.

The OsIAA10/OsTIR1 interaction is expected to lead to degradation of OsIAA10 by the ubiquitin-26S proteasome [24]. Thus, we tested whether disruption of the OsIAA10/OsTIR1 interaction by P2 leads to inhibition of OsIAA10 degradation and hence its stabilization. This was first assessed in a cell-free assay in which MBP-OsIAA10 was incubated in protein extracts prepared from RDV-infected or mock-inoculated rice plants. The half-life of MBP-OsIAA10 more than doubled in the RDV-infected extracts (Fig 3D), suggesting attenuation of OsIAA10 degradation following RDV-infection. This P2-mediated inhibition of degradation is specific to OsIAA10, because degradation of OsIAA1, which does not interact with P2, was not affected by P2 (S4 Fig). We further confirmed this attenuation in cell-free extracts prepared from *N*. *benthamiana* leaves transiently expressing HA-P2, which did not occur in extracts from leaves expressing an empty vector (mock) (Fig 3E). Consistent with the *in vitro* results, the OsIAA10 protein accumulated to significantly higher levels in RDV-infected rice plants compared to mock-inoculated wild-type plants (Fig 3F), despite the fact that its mRNA levels, as measured by qPCR were mostly unchanged by RDV infection (Fig 3G and S5 Fig). To test if P2 inhibits OsIAA10 degradation *in vivo*, we co-expressed FLAG-OsIAA10 and HA-P2 in *N*. *benthamiana* leaves. An HA-P2Δ (1–90)-expressing construct was used as a negative control. In addition, we included a β-glucuronidase (GUSA)-expressing construct to ensure that protein translation occurred in all of the samples at similar levels. The results showed that the FLAG-OsIAA10 protein accumulated to higher levels in the presence of full-length P2, but not in the presence of the P2Δ(1–90) mutant (Fig 3H), suggesting that the P2/OsIAA10 interaction caused stabilization of OsIAA10. Together these data provide molecular and biochemical evidence consistent with the interpretation that P2 manipulates the initiation of auxin signaling by shielding OsIAA10 from OsTIR1-mediated degradation.

### Rice plants overexpressing a degradation-resistant OsIAA10 mutant phenocopy the symptoms of RDV-infected rice

**Fig 4:**
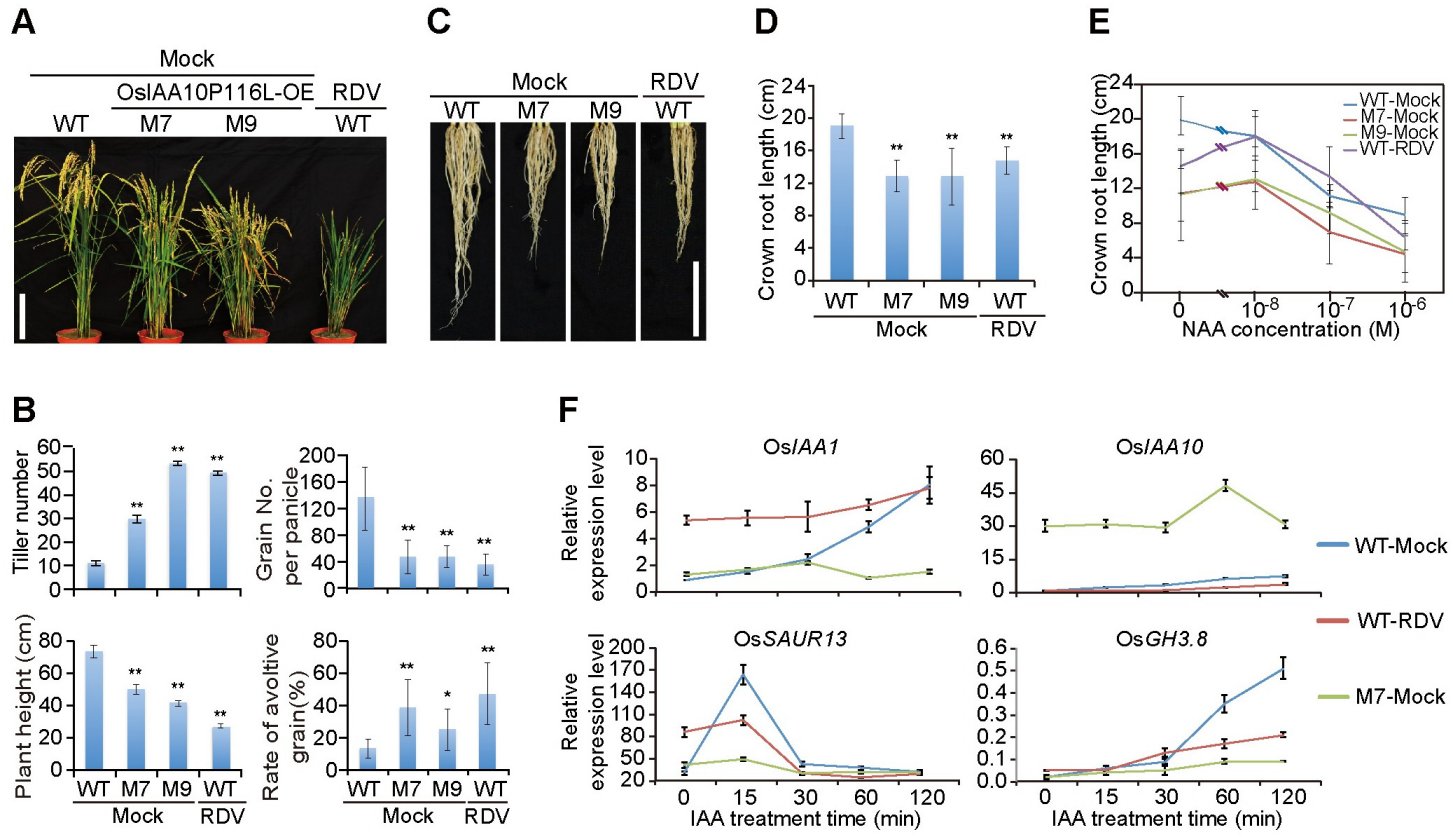
OsIAA10P116L-overexpressing transgenic rice plants phenocopy RDV-infected rice plants. (A) Morphologies of mock-inoculated M7 and M9 as well as WT-RDV plants at maturity stage. Bar: 15 cm. (B) Schematic representation of the tiller number, plant height, grain number per panicle, and rate of avoltive grain (%) for the above plants at maturity stage. The average (±SD) values were obtained from three biological repeats, with 15 plants from each line in every repeat. Significant differences were indicated (*P<0.05, **P<0.01) based on Student’s *t*-test. (C and D) Phenotypes (C) and schematic representation (D) of the length of the crown roots of 6-week-old mock-inoculated WT and OsIAA10P116L-overexpressing (M7 and M9), as well as RDV-infected WT, rice plants. Bar: 10 cm. The average (±SD) values were obtained from three biological repeats, with 15 plants from each line in every repeat. Significant differences were indicated (**P<0.01) based on Student’s *t*-test. (E) Lengths of crown roots of 6-week-old mock-inoculated WT, M7 and M9 as well as RDV-infected WT rice seedlings cultured in a liquid nutrient containing the indicated concentration of NAA. The average (±SD) values were obtained from three biological repeats, with 15 plants from each line in every repeat. (F) qPCR analysis of auxin-induced gene expression after IAA treatment in mock-inoculated WT and M7 as well as RDV-infected WT rice seedlings. The expression levels were normalized using the signal from *OsEF1a*, and values are mean ± SD (n = 3 biological replicates).

To determine the biological significance of OsIAA10 stabilization by the RDV P2 protein, we generated a degradation-resistant OsIAA10 mutant by altering the highly conserved proline residue at position 116 (the second P of the 13 conserved aa within domain II) to a leucine (S6A Fig) [31]. This OsIAA10P116L mutant, although still bound to P2, failed to interact with OsTIR1 in yeast (S6B Fig), and hence became resistant to degradation mediated by the ubiquitin ligase, SCF^OsTIR1^, in the cell-free assays (S6C Fig). Consistent with an active role of OsIAA10 in auxin signaling in rice, transgenic rice plants overexpressing OsIAA10P116L were substantially stunted compared to non-transgenic control plants or transgenic plants overexpressing the wild-type OsIAA10 (S6D Fig). Notably, these transgenic plants also developed more tillers, shorter crown roots and lower fertility rates, strikingly similar to the symptoms exhibited in RDV-infected rice plants (Fig 4A–4E, S7 and S8 Figs). Overexpression of wild-type OsIAA10 caused a moderately increased tillering and dwarf phenotypes (S6D and S7 Figs). The severity of which correlated with the accumulation levels of the OsIAA10 (or OsIAA10P116L) (S6D Fig). Importantly, OsIAA10P116L-transgenic plants also exhibited insensitivity to auxin treatment typical of RDV-infected plants, as measured by crown root elongation under different NAA concentrations (Fig 4E and S8 Fig). Specifically, as expected, treatment with 10^−8^ M NAA caused inhibition of root elongation in mock-inoculated wild-type plants (Fig 4E). In contrast, 10^−7^ M NAA was required to trigger similar inhibition in the OsIAA10P116L-overexpressing M7 and M9 plant lines or RDV-infected rice plants (Fig 4E). Furthermore, qPCR results showed that the expression of auxin-inducible genes was severely diminished in both M7 seedlings and RDV-infected plants (Fig 4F and S9 Fig). Together, these data suggest that the collective symptoms of RDV infection, including dwarfism, increased tiller number, shorter crown roots, and decreased seed fertility, are adequately explained by RDV P2-mediated stabilization of OsIAA10, which in turn leads to auxin-resistant phenotypes reminiscent of the OsIAA10 gain-of-function mutant.

### OsIAA10 stabilization is needed for optimal RDV infectivity

**Fig 5:**
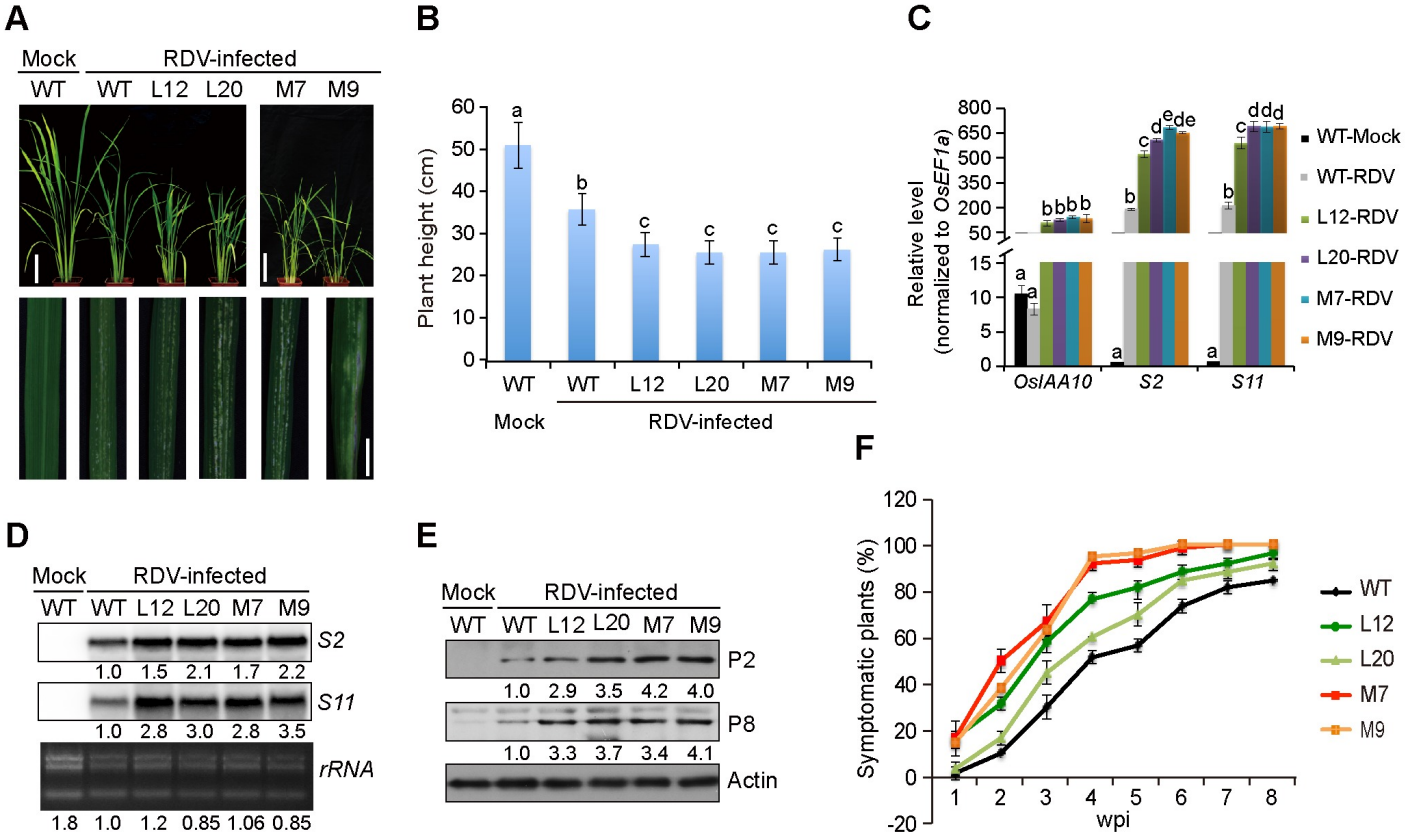
OsIAA10 accumulation enhances RDV pathogenicity. (A) Phenotypes of RDV-infected WT, L12, L20, M7, and M9 rice plants. Photos were taken 4 weeks after RDV inoculation. The areas of white specks on the leaves represent the degree of disease symptoms. Scale bars: 10 cm (upper panel) and 1 cm (lower panel). (B) Schematic representation of plant height for the plants in (A). The average (±SD) values were obtained from three biological repeats, with 15 plants from each line in every repeat. Different letters indicate significant differences (*p* < 0.05) based on the Tukey-Kramer HSD test. (C) qRT-PCR assay showing the relative expression level of *OsIAA10* and RDV RNAs (*S2* and *S11*) in plants in (A). The expression levels were normalized using the signal from *OsEF1a*, and values are mean ± SD (n = 3 biological replicates). Different letters indicate significant differences (*p* < 0.05) based on the Tukey-Kramer HSD test. (D and E) Northern (D) and Western (E) blots showing the accumulation of RDV RNAs and proteins in RDV-infected WT, L12, L20, M7 and M9 rice lines. rRNAs were used as a loading control for RNA and Actin was used as a loading control for proteins. (F) Time course of symptomatic plants (%) of WT, L12, L20, M7 and M9 from one week-post-inoculation (wpi) to 8 wpi. Inoculation assays were repeated three times, respectively. The error bars indicate SD. L12 and L20 are transgenic rice lines overexpressing OsIAA10; M7 and M9 are transgenic rice lines overexpressing OsIAA10P116L.

Next, we determined whether the process of RDV infection is affected by P2-mediated stabilization of OsIAA10 protein and/or subsequent manipulation of auxin signaling. To this end, we subjected transgenic plants overexpressing wild-type OsIAA10 (lines L12 and L20) and OsIAA10P116L (lines M7 and M9) to RDV inoculation via feeding by the viruliferous insect vector *N*. *cincticeps*. Specifically, two-week-old seedlings were exposed to the viruliferous leafhoppers for two days, during which time, the number of insects settling on each particular type of plants was recorded twice a day and used to calculate the index of non-preference for each line [49]. The insects did not show a preference for a particular type of plants (S1 Table). The insects were removed after the 2-day feeding period, the infected plants were monitored on a daily basis, and the percentage of plants showing RDV symptoms was recorded once a week since the date the insects were removed. The earliest symptom evident on the systemic leaves of RDV-infected wild-type rice plants is the appearance of tiny chlorotic spots commonly referred to as specks [50]. We found that the speck size dramatically increased on the leaves of transgenic L12, L20, M7, and M9 plants, frequently becoming chlorotic strips (Fig 5A). RDV-induced stunting was also more pronounced in the transgenic plants (Fig 5A and 5B). Therefore, increased accumulation of the OsIAA10 protein correlated with more severe disease symptoms, suggesting that its stabilization of OsIAA10 by RDV P2 leads to more rigorous infections.

In agreement with the intensified RDV symptoms, the symptomatic transgenic L12, L20, M7, and M9 lines accumulated higher levels of RDV genomic RNAs (Fig 5C and 5D) and RNA-encoded proteins (Fig 5E). In addition, the percentage of plants with RDV symptoms recorded over an 8-week period was consistently higher in transgenic plants than in wild-type control plants (Fig 5F). These data demonstrate that higher levels of OsIAA10 protein correlated with more rigorous RDV infection, strongly suggesting that the RDV P2/OsIAA10 interaction, and possibly the resulting disruption of auxin signaling, confers a selective advantage to RDV multiplication.

**Fig 6:**
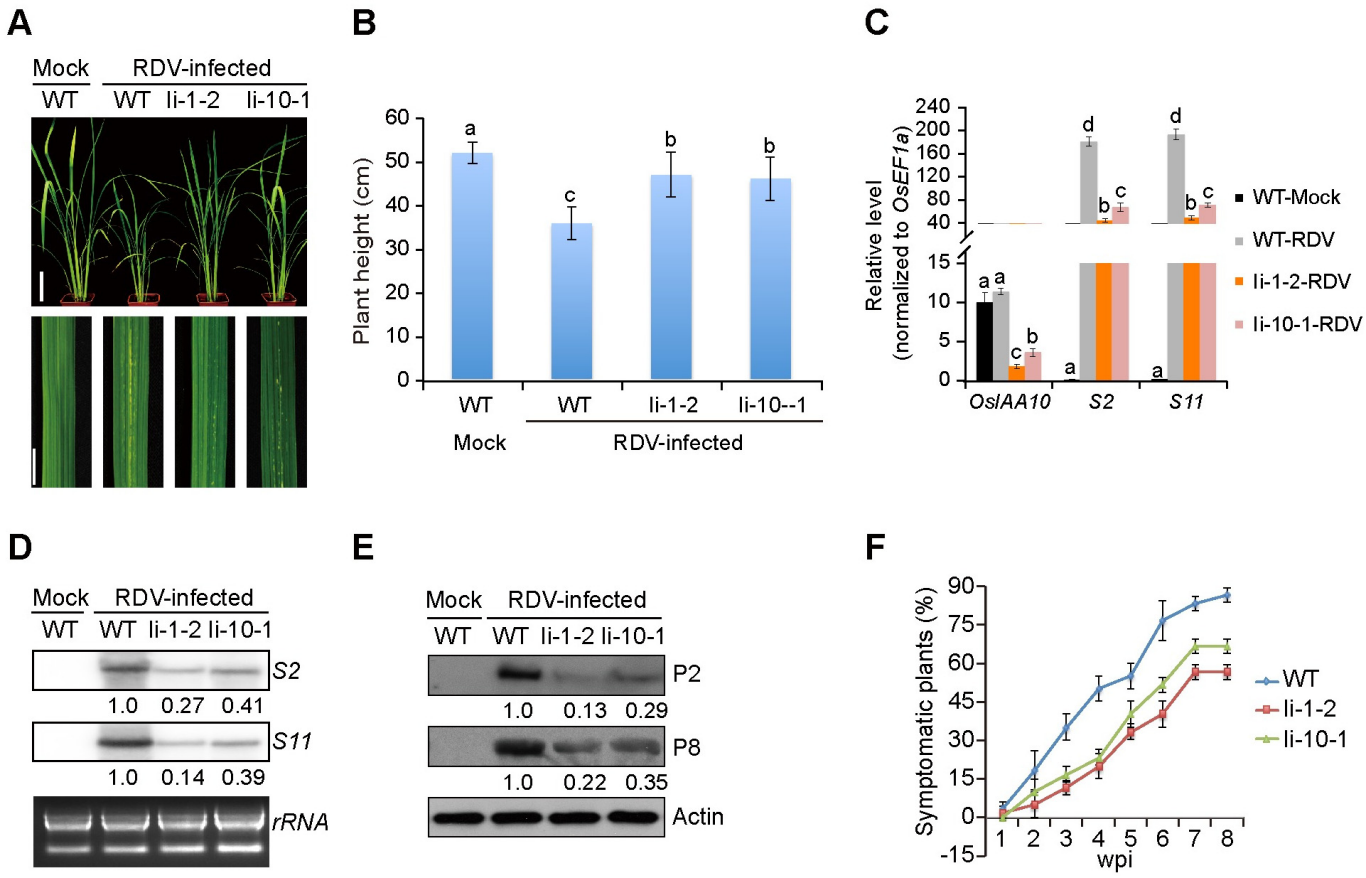
Reduced expression of OsIAA10 inhibits RDV infection and replication. (A) Phenotypes of RDV-infected WT, Ii-1-2, and Ii-10-1 rice plants. Photos were taken 4 weeks after RDV-inoculation. The areas of white specks on the leaves represent the degree of disease symptoms. Scale bars: 10 cm (upper panel) and 1 cm (lower panel). (B) Schematic representation of plant height for the plants in (A). The average (±SD) values were obtained from three biological repeats, with 15 plants from each line in every repeat. Different letters indicate significant differences (*p* < 0.05) based on the Tukey-Kramer HSD test. (C) qRT-PCR assay showing the relative expression level of *OsIAA10* and RDV RNAs (*S2* and *S11*) in plants in (A). The expression levels were normalized using the signal from *OsEF1a*, and values are mean ± SD (n = 3 biological replicates). Different letters indicate significant differences (*p* < 0.05) based on the Tukey-Kramer HSD test. (D and E) Northern (D) and Western (E) blotting showing the accumulation of RDV RNAs and proteins in the corresponding rice lines in *A*. rRNAs were used as a loading control for RNA and Actin was used as a loading control for proteins. (F) Time course of RDV symptomatic plants (%) in WT, Ii-1-2 and Ii-10-1 rice lines from one week-post-inoculation (wpi) to 8 wpi. Inoculation assays were repeated three times, respectively. The error bars indicate SD. Ii-1-2 and Ii-10-1 are OsIAA10RNAi transgenic rice lines.

If OsIAA10 stabilization directly benefits RDV infection, decreasing its expression should have the opposite effects. To test this hypothesis, we knocked down OsIAA10 expression in transgenic rice plants using dsRNA derived from the entire coding region of OsIAA10. More than 10 independent RNA interference (RNAi) lines (referred to as Ii lines), were obtained, and the significant reduction in OsIAA10 expression was confirmed by qPCR (S10A Fig). There was no obvious difference in growth between the Ii and wild-type rice lines (S10B Fig), and the leafhoppers showed no preference for either plant (S1 Table). However, RDV-infected Ii-1-2 and Ii-10-1 plants displayed weaker symptoms than RDV-infected wild-type plants, such as less dwarfism, tillers, and specks (Fig 6A and 6B). Likewise, there was less accumulation of RDV genomic RNAs (Fig 6C and 6D) and proteins (Fig 6E) in the infected Ii-1-2 and Ii-10-1 rice lines, as well as a lower percentage of infected transgenic plants (Fig 6F). Thus, knockdown of OsIAA10 negatively affects RDV infection, demonstrating its critical role in facilitating RDV infection, replication and symptom development.

## Discussion

**Fig 7:**
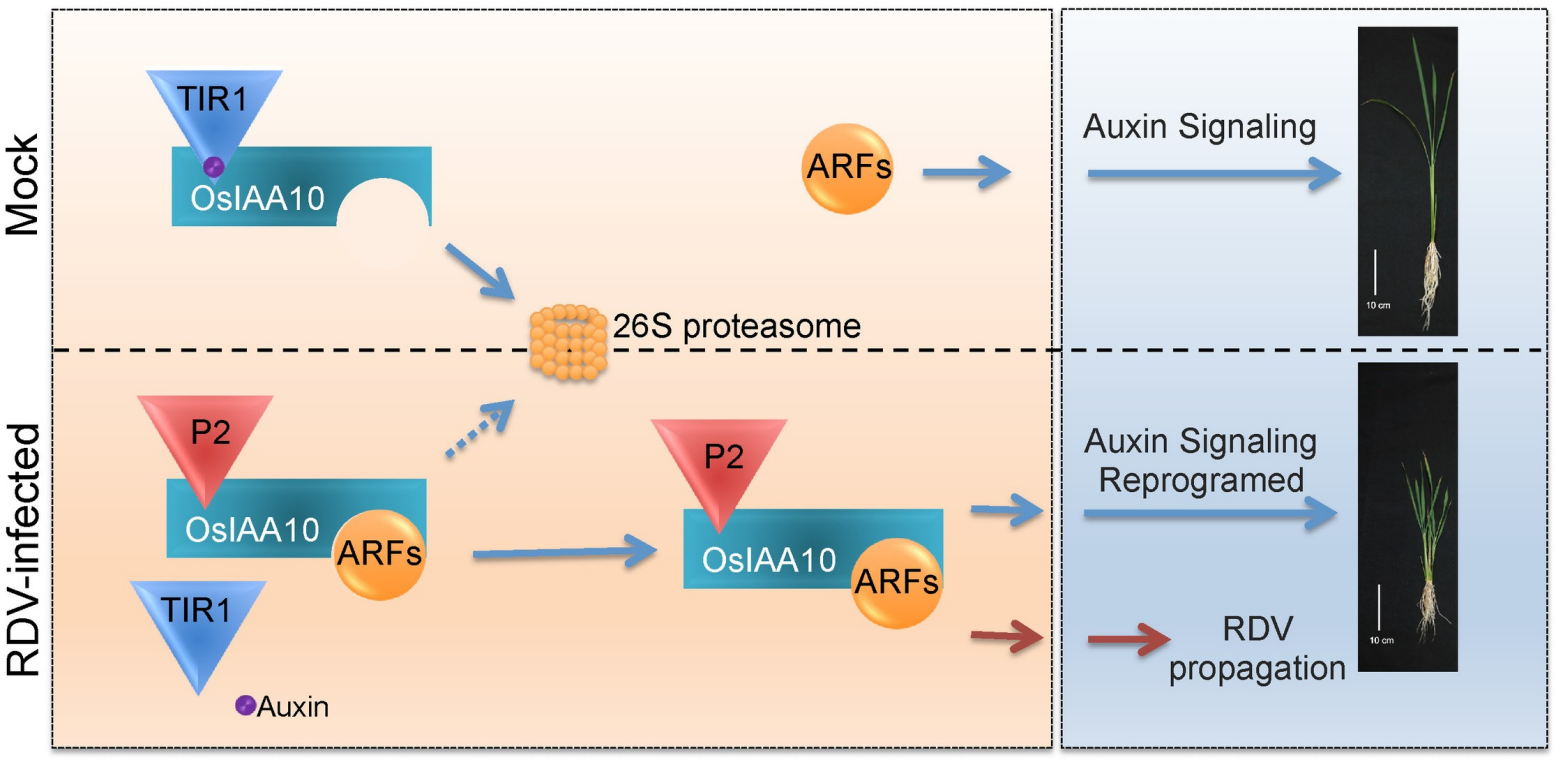
Proposed model. In mock rice plants, high auxin concentration promotes the interaction between OsIAA10 and OsTIR1, leading to the ubiquitination and 26S proteasome degradation of OsIAA10, releasing the specific OsARFs. Then genes of auxin signaling pathway were adequately regulated by corresponding ARF transcription factors, promoting normal rice growth. Under RDV infection condition, P2 binds to the domain II of OsIAA10, blocking its association with OsTIR1 for degradation. The stabilized OsIAA10 binds to corresponding OsARFs, manipulating down-stream gene expression in the auxin signaling pathway. Reprogramed auxin signaling causes stunting, more tillering, shorter crown roots to the rice plants and promotes RDV propagation in rice.

In the current study, we demonstrate a novel mechanism through which the initiation of auxin signaling pathways is reprogrammed by a viral protein, thereby causing morphogenesis alterations in rice and enhancing the viral infection (for a model, see Fig 7). Biochemically, this has evolved to be a highly specific process in which the RDV P2 protein interacts with OsIAA10, but not with other tested Aux/IAA proteins; and P2 binds domain II of OsIAA10, thereby inhibiting its interaction with SCF^TIR1/AFBs^ and subsequent degradation that is key for the regulation of normal growth and development. Biologically, this reprogramming of auxin signaling initiation leads to the abnormal expression of rice genes that apparently benefit viral infection and enhance disease symptoms. The importance of this new mechanism, from a practical point of view and as demonstrated here, is that the engineered reduction of OsIAA10 can improve the resistance of rice to RDV infection. RDV-mediated alterations in the auxin signaling pathway are not just limited to OsIAA10, the mRNA expression of downstream genes with auxin response elements (AuxREs), including genes from the Aux/IAA, GH3, and SAUR families, were directly or indirectly affected by RDV infection (Fig 1E). However, OsIAA10 protein levels but not mRNA levels were changed upon RDV infection (Figs 1E, 3G, and S5 Fig).

Previous studies have shown that the tobacco mosaic virus (TMV) replicase protein interacts with several Aux/IAA proteins, including AtIAA26, to prevent their nuclear localization, resulting in transcriptional reprogramming of a large number of auxin-responsive genes [7–9]. In our study, the RDV P2 protein interacted with OsIAA10 (Fig 2), and RDV infection manipulated the auxin pathway in rice (Fig 1). To determine if RDV causes alterations in the auxin signaling pathway by the same mechanism as the TMV, we examined the subcellular localization of OsIAA10 and found that it was localized in both the nucleus and cytoplast when transiently expressed in the tobacco leaf alone or together with P2 (S11 Fig); P2 does not affect its nuclear localization (S11 Fig). The interaction of P2 with OsIAA10 does not change the sub-cellular localization and furthermore, P2 interacted with domain II of OsIAA10 (Fig 2D and S2D Fig) whereas OsARFs and Aux/IAA proteins form heterodimers with their PB1 domains [20,22–30]. Taking these data together, we presume that the interaction between P2 and OsIAA10 would not inhibit the binding of OsIAA10 to OsARFs, and thus exclude the possibility that P2 prevents OsIAA10 from interacting with ARFs by sequestering it outside the nucleus, as the TMV 126K protein does with its interacting Aux/IAA proteins. We also showed that P2/OsIAA10 interaction prevented OsTIR1 from accessing OsIAA10 by occupying the same interaction domain (Figs 2D and 3.). Thus our work shows a novel mechanism underlying viral protein interference with auxin signaling.

There are 31 Aux/IAA proteins in rice [21]. Phylogenetic analysis of the full-length rice Aux/IAA proteins sequences showed that the overall identities range from 14% to 76%. However, identities of the aa sequences within the four conserved domains reaches up to 96% [21]. Protein from plants like LRT2 [51] and pathogen component or protein like the TMV replicase [8] are able to interact with most or several Aux/IAA proteins. In our yeast two-hybrid system, P2 only interacted with OsIAA10, but not with the other 19 family members tested (S1C Fig), possibly because P2 interacts with domain II of OsIAA10 (aa 91–141) (Fig 2D), including the conserved portion (16 aas) and the left flanking domain (S2A and S12 Figs). The aa sequence identity in the conserved domains of the rice Aux/IAA proteins are very conserved and reach up to 96%, but significantly varied in the left flanking domain, suggesting that the left flanking domain may play an important role in the P2/OsIAA10-specific interaction. In addition, the domains or sections for OsIAA10 to interact with P2 and OsTIR1 are very close, but the sites for each specific interaction are different because the OsIAA10P116L mutant still interacts with P2, but not with OsTIR1 (S6A Fig). These results suggest that the interaction of P2 with OsIAA10 may shield the site in OsIAA10 that is necessary for OsTIR1 binding.

Stabilization of OsIAA10 causes phenotypes similar to that of the RDV symptoms such as stunted growth, increased tiller number, shorter crown roots, and lower fertility rate (Fig 4). Previously findings in our laboratory showed that the dwarf symptom resulting from RDV infection is due to the interaction between P2 and *β*-*ent*-kaurene oxidases, which catalyze *ent*-kaurene to *ent*-kaurene acid in gibberellic acid synthesis [10]. This interaction caused significant decreased levels of gibberellic acid (GA) in RDV-infected plants [10]. However, the crosstalk involved to produce disease is unknown. To address this issue, we infected a natural rice mutant of *β*-*ent*-kaurene oxidases, named *d35*, which contains a single nucleotide substitution located in exon 5 of OsKO2 resulting in a replacement of an arginine by serine and a semi-dwarf phenotype [52]. The infection of *d35* by RDV caused even more severe stunting of this rice mutant (S13 Fig). These results suggest that another pathway also causes the dwarf phenotype upon RDV infection, such as manipulation of auxin signaling by P2, as shown in this study.

Different host plant-virus combinations may affect auxin homeostasis differently, and vice versa. Auxin signaling would be modulated to either promote or disrupt virus biology [4]. The results from this study show that auxin signaling plays an active role in antiviral defense that needs to be repressed by RDV in order to attain productive infection. Consistent with this idea, it was previously shown that TMV replicase protein interacts with several Aux/IAA proteins to prevent their nuclear localization, leading to transcriptional reprogramming of auxin-responsive genes that favors viral multiplication and symptom development [7–9]. Likewise, the external application of IAA reduced the replication of a different virus, namely, the white clover mosaic virus [53], further corroborating the antiviral role of auxin signaling. Therefore, modulation or reprogramming of auxin signaling is a key factor in the successful infection for many viruses. While this remains to be an interesting, but poorly understood phenomenon, the existing literature does provide some clues. Viruses are intracellular pathogens that need to cross cellular boundaries to spread through the entire infected plant. Enhanced auxin signaling could accelerate the division of primordial cells, allowing the host to outcompete viruses, thereby keeping a fraction of dividing cells virus-free [39]. Additionally, auxin signaling may also activate the expression of certain antiviral defense genes. It was reported that in IAA26-stablized *A*. *thaliana* plants, TMV infection and accumulation were inhibited, and defense response genes were induced [54]. In this study, OsIAA10-stablized rice plants were more hypersensitive to RDV infection, and the defense response genes, such as *PR2*, *PR10*, *JAZ12*, *WRKY13* and *WRKY45*, were downregulated (S14 Fig).

The reprogramming of auxin signaling initiation by P2, together with P2 inhibition of GA synthesis via interaction with rice *ent*-kaurene oxidases [10], demonstrate the broad significance of viral inhibition or manipulation of multiple hormonal pathways to benefit infection and enhance disease symptoms. Importantly, P2 is targeted for degradation by the rice protein OsRFPH2-10, a RING FINGER type E3 ubiquitin ligase [50]. In light of these findings, we propose that the RDV P2 protein interacts with diverse rice proteins to inhibit plant hormone biosynthesis or to reprogram hormone signaling, and that OsRFPH2-10 interactions with P2 to promote its degradation, represent a new paradigm of arms race between viruses and their hosts, one of the defining factors of evolution, and provide information on the mechanism of viral protein reprogramming, which may be extrapolated to other microbe-plant interaction systems.

## Materials and Methods

### Plant growth and virus inoculation

Rice plant growth conditions and virus inoculation methods were as previously described [55]. Briefly, rice plants (cv. Zhonghua11) seedlings were grown in a greenhouse at 28–30°C and 60%±5% relative humidity under natural sunlight. For viral infection, plants were exposed to the viruliferous (RDV-carrying) or virus-free (mock) insects of leafhoppers (*N*. *cincticeps*) at a ratio of 1:2 (two insects per plant) for 48 h when they were about 2 weeks old. During the 2-day infection periods, the number of insects settling on each row of plants was counted twice a day for 2 days. The mean number of settled insects on each seedling was used as the index of non-preference for each line (S1 Table). Details of the procedure have been previously described [49]. Then the insects were removed, and the plants were grown in the greenhouse under the same conditions as described above. The inoculated plants were monitored daily for the appearance of viral symptoms. The numbers of rice with symptoms for each line were recorded every week (S2 and S3 Tables). Rice plants for phenotype analysis were transplanted to an isolated paddy field when they were 4 weeks old. The height of the adult plants was determined by measuring the length of the main tiller of each transgenic plant from the top of the 1^st^ internode to the bottom of the last internode.

### DNA constructs

All of the PCR products used for cloning were generated using KOD DNA polymerase (TOYOBO, Osaka, Japan). Plasmids and PCR primers for the PCR experiments are listed in S4 and S5 Tables. All plasmids were sequenced from both ends for confirmation.

### Generation and characterization of transgenic rice

*Oryza sativa japonica* cv. ZH11 was transformed with constructs *35S*:*FLAG -OsIAA10*, *35S*:*FLAG-OsIAA10P116L* and *Actin*:*OsIAA10RNAi*, respectively, with *Agrobacterium tumefaciens*-mediated transformation at Weiming Kaituo Co., Ltd (Beijing, China). The overexpression lines were characterized by Western blotting and OsIAA10RNAi lines were characterized by qPCR. The T2 transgenic lines that stably maintained the transgenes were chosen for phenotype analyses and viral infection assays.

### Yeast two-hybrid assay

Yeast transformation and screening were performed according to the manufacturer’s instructions (Clontech; Mountain View, CA, USA). Yeast AH109 cells were co-transformed with specific bait and prey constructs. All of the yeast transformants were grown on an SD/-Leu/-Trp/-His/-Ade (SD-L-W-H-Ade) medium for selection or the interaction test.

### *Agrobacterium*-mediated transient expression, protein extraction, and Western blot analysis

The *A*. *tumefaciens* strain EHA105 was used in these experiments. The procedures used for agrobacterial infiltration, protein extraction, and Western blotting were as previously described [10,50]. The following antibodies were purchased from commercial sources: anti-HA (TIANGEN; Beijing, China), anti-HA-peroxidase (Roche; Mannheim, Germany), anti-FLAG-peroxidase (Sigma; St Louis, MO, USA), anti-FLAG (Sigma), anti-β-GUS (Sigma), anti-actin (EASYBIO; Beijing, China), anti-MBP (Sigma), and anti-GFP (MBL; Nagoya, Japan). The anti-OsIAA10, anti-P2, and anti-P8 antibodies were produced in our laboratory.

### Co-IP

Co-IP from rice cells and *N*. *benthamiana* cells were performed as described in Liu et al. (2014). Briefly, samples were extracted with IP buffer (50 mM Tris-HCl pH7.5, 150 mM NaCl, 0.1% NP-40, 5 mM DTT, protease inhibitor cocktail Complete Mini tablets [Roche]) as previously described [10]. Relevant antibodies were added to the cell lysates (10 μg ml^-1^) and MG132 (Sigma) was added at a final concentration of 50 μM to prevent protein degradation. The mixtures were kept at 4°C with gentle shaking for 30 min. The IP complex was captured by adding 20 μl ml^-1^ rec-protein G-Sepharose 4B Conjugate (Invitrogen; Carlsbad, CA, USA), following by shaking at 4°C for another 1 h. The Sepharose beads were recovered by centrifugation at 1000×g for 30 sec and washing three times with cold TBS (50 mM Tris-HCl pH7.5, 150 mM NaCl). Then 20 μl of sample buffer (50 mM Tris-HCl pH6.8, 2% SDS, 6% Glycerol, 0.1M DTT, 0.02% bromophenol blue) was added into the beads. After boiling for 10 min and centrifugation, the samples were loaded onto the SDS-PAGE gels for Western blotting analysis.

### LCI assay

LCI assays were performed as previously described [56]. All of the related constructs were transformed into *A*. *tumefaciens* strain EHA105. An equal volume of *A*. *tumefaciens* harboring pCAMBIA-nLUC and pCAMBIA-cLUC (or their derivative constructs) were mixed to a final concentration of OD_600_ = 1.0. Four different combinations of *A*. *tumefaciens* were infiltrated into four different positions in the same leaves of *Nicotiana benthamiana*. After 3 days, 30 min before detection, 0.2 mM luciferin (Promega; Madison, WI, USA) was infiltrated into the same positions that *A*. *tumefaciens* infiltrated. Then luciferase activity was measured with a low-light cooled CCD imaging apparatus (NightOWL II LB983 with indiGO software).

### RNA extraction, semi-quantitative reverse transcription-PCR, and qPCR

RNA was extracted with TRIzol Reagent (Invitrogen) according to the manufacturer’s instructions. The RNAs used for reverse transcription (RT)-PCR and qPCR were treated with RQ1 RNase-free DNase (Promega) to remove DNA. A concentration of 2 μg total RNA was reverse transcribed with the SuperScript III Reverse Transcriptase kit (Invitrogen) using oligo (dT) primers according to the manufacturer’s instructions. RT-PCR assays were performed with 2×Taq Polymerase Mix (Kangwei Shiji; Beijing, China) and qPCR reactions were performed using the SYBR Green Real-Time PCR Master Mix (TOYOBO) following the manufacturer’s instructions. Relative transcript levels were calculated using the 2^-ΔΔC(t)^ method with the OsEF1a transcript serving as the internal standards. Each data set was derived from at least three biological repeats. The primers are listed in S6 Table.

### Northern blot analysis

Total RNA was obtained from rice plants as described above. Northern blot analysis was performed as previously described [50]. RNA (8 μg per lane) was separated by electrophoresis on 1.2% (w/v) formaldehyde-denaturing agarose gels and blotted onto N+ nylon membrane (Amersham; Buckinghamshire, UK). The probes were amplified from PCR products with primers listed in S5 Table, and labeled using α-^32^P-dCTP with the Random Primer DNA Labeling Kit (TaKaRa; Shiga, Japan). Hybridization and detection were performed according to instructions that came with the PerfectHybTm Plus Hybridization Buffer (Sigma).

### *In vitro* pull-down assay

*In vitro* pull-down and competitive pull-down assays were carried out as previously described [57]. Constructs pMAL-p2x-OsIAA10, pMAL-p2x-OsIAA10P116L, pMAL-p2x-OsTIR1, pMAL-p2x-P2(1–786), pGEX-4T-1-OsIAA10, and pCST-P2(1–786) constructs, as well as empty pMAL-p2x and pGEX-4T-1 vectors were individually transformed into *Escherichia coli* Transetta (DE3) (Transgene; Beijing, China). Protein expression was induced by isopropyl-β-D-thiogalactoside (IPTG). Soluble MBP fusion proteins were extracted and immobilized onto amylose resin (New England Biolabs; Ipswich, MA, USA). Soluble GST fusion proteins were extracted and immobilized onto glutathione sepharose beads (GE Healthcare; Little Chalfont, Buckinghamshire, UK). For competitive pull-down assays, 3 μg of GST-OsIAA10 with 0, 3, 6, or 12 μg GST-P2(1–786) or 6 μg of GST alone were incubated with immobilized MBP-OsTIR1 (6 μg) at 4°C for 1 h. Proteins retained on the beads were resolved by SDS-PAGE and detected with anti-GST or anti-MBP antibodies, respectively.

### MST assays

The MST assay was performed as previously described [47,48,58]. MBP-OsTIR1 proteins were labeled with the red fluorescent dye NHS according to the Monolith NT^™^ Protein Labeling Kit RED-NHS instructions (NanoTemper Technologies GmbH; München, Germany). In OsTIR1/OsIAA10 interaction assays, the concentration of NHS-labeled OsTIR1 was kept constant at 75 nM and that of NAA was kept constant at 2.5 μM whereas the concentrations of OsIAA10 were gradient-diluted (80,000 nM, 40,000 nM, 20,000 nM until 1.44 nM). After a short incubation, the samples were loaded into MST standard treated glass capillaries. Measurements were performed at 25°C in buffer containing 20 mM Tris, pH 8.0 and 150 mM NaCl, using 30% LED power and 20% MST power. The assays were repeated three times for each affinity measurement. Data analyses were performed using the Nanotemper Analysis and OriginPro 8.0 software provided by the manufacturer. In competitive interaction assays, 2.5 μM of MBP-P2 or MBP was added to 75 nM NHS-labeled-OsTIR1, 2.5 μM of NAA and gradient-diluted concentrations of OsIAA10. After a short incubation, the samples were loaded into MST standard treated glass capillaries for MST analysis as described above.

### Cell-free protein degradation assays

Cell-free protein degradation assays were performed as previously described [59]. Briefly, fresh total protein extracts were prepared from 0.5 g of rice or *N*. *benthamiana* leaves in 1 mL cell-free degradation buffer (25 mM Tris-HCl, pH 7.5, 10 mM NaCl, 10 mM MgCl2, 4 mM PMSF, 5 mM DTT, and 10 mM ATP) to establish the cell-free degradation system. Then 100 μg of MBP-OsIAA10, MBP-OsIAA10P116L, or MBP-OsIAA1 was added into the system. For the 26S proteasome degradation assay test, MG132 (final concentration of 50 μM, dissolved in DMSO) or DMSO was added into the test system or control system, respectively. Degradation assays with the rice extracts were incubated at room temperature for 120 min, and samples were collected at 0, 5, 15, 30, 60, and 120 min for standard immunoblot assays as described above. Degradation assays with *N*. *benthamiana* extracts were incubated at 4°C for 180 min, and samples were collected at 0, 20, 40, 80, and 180 min for standard immunoblot assays. The relative level of the remaining protein in each lane was calculated using ImageJ software.

### *In vivo* degradation assay

*In vivo* protein degradation experiments (degradation assay in tobacco) were carried out as previously described [60]. *A*. *tumefaciens* strains containing *pCambia1301-FLAG-OsIAA10* (because the GUSA sequence is in the vector pCambia1301 backbone, so *pCambia1301-FLAG-OsIAA10* can express both OsIAA10 and GUSA proteins) and *pWM101-HAS2* (which express HAP2) or *pWM101-HAS2Δ(1–270)* (which express HAP2Δ(1–90)) or *pWM101* vector were mixed at a ratio of 1:2 to a final concentration of OD_600_ = 1. These three different combinations were infiltrated into the *N*. *benthamiana* leaves. After 3 days, 4 hours before harvesting the sample, 50 μM IAA was infiltrated into the same leaves. Then the leaves infiltrated with different combinations were sampled separately and grinded in liquid nitrogen for protein and RNA extractions.

### Auxin response assays

For assays on auxin inhibition of root elongation, 4-week-old rice plants of wild-type (WT), WT-RDV, M7, and M9 were cultured in a liquid rice culture solution [32] supplemented with different concentrations of NAA. After 10 days, the root phenotypes were photographed using a digital camera and the root lengths were measured. To analyze induction of auxin-responsive genes, leaves of 2-month-old WT, WT-RDV, and M7 plants were cut into 0.5–1 cm pieces, immersed into the liquid rice culture solution with 20 μM IAA, and sampled at 0, 15, 30, 60, and 120 min for isolation of the total RNA.

## Supporting Information

S1 Fig RDV P2 specifically interacts with OsIAA10. (A) Co-immunoprecipitation assays showing P2-OsIAA10 interaction in *N*. *benthamiana* leaves. (B) Phylogenetic relationship among the rice Aux/IAA proteins. The unrooted tree was generated using ClustalX program by neighbor-joining method. Bootstrap values form 100 replicates are indicated at each node. (C) Among the 20 rice Aux/IAA proteins tested, only OsIAA10 interacts with P2 in yeast two-hybrid assays. Yeast transformants were spotted on the control medium (SD-L-W) and selection medium (SD-L-W-H-Ade). (D) OsIAA10 specifically interacts with P2, but not other RDV proteins.
(TIF)

S2 Fig OsIAA10 is a functional Aux/IAA protein. (A) OsIAA10 contains the four conserved domains (underlined), shared with the other Aux/IAA family members. (B) Cell-free degradation assays of MBP-OsIAA10 in the presence or absence of MG132 in rice extracts. +MG132: final MG132 concentration of 50 μM; -MG132: equal volume of DMSO (MG132 solvent) as a negative control. Samples were collected at 0, 5, 15, 30, 60, and 120 min after incubation at room temperature. Rubisco Large protein (RuL) was used as a loading control for total plant proteins. RL: relative level of remaining MBP-OsIAA10 protein. On the right are normalized plots for the degradation data of MBP-OsIAA10 shown on the left. (C) OsIAA10 interacts with OsTIR1 in the presence of auxin. Phylogenetic analysis of rice TIR1-like proteins is shown on the left. The unrooted tree was generated using ClustalX program by neighbor-joining method. Bootstrap values (above 50%) from 100 replicates are indicated at each node. The right panels show interaction or no interaction of OsIAA10 with OsTIR1 and other OsTIR1-like proteins in yeast. Yeast transformants were spotted on the selection medium (SD-L-W-H-Ade) with or without 50 μM IAA. (D) OsIAA10 domain II interacts with OsTIR1 in yeast.
(TIF)

S3 Fig P2 does not interact with OsTIR1. Yeast two-hybrid assays show that OsTIR1 does not interact with P2. Yeast transformants were spotted on the control medium (SD-L-W) and selection medium (SD-L-W-H-Ade) with or without 50 μM IAA.
(TIF)

S4 Fig P2 does not affect OsIAA1 degradation. (A) Degradation of OsIAA1 is 26S proteasome-dependent. Cell-free degradation assays of MBP-OsIAA1 in the presence or absence of MG132 in rice extracts. +MG132, final MG132 concentration of 50 μM; -MG132, equal volume of DMSO (MG132 solvent) as a negative control. (B) Cell-free degradation assays of MBP-OsIAA1 in mock or RDV-infected rice extracts. Samples were collected at 0, 5, 15, 30, 60 and 120 min after incubation at room temperature. Rubisco Large protein (RuL) was used as a loading control of total plant protein. RL: relative level of remaining MBP-OsIAA1 protein.
(TIF)

S5 Fig RDV infection does not alter the transcription levels of *OsIAA10*. (A) Relative expression levels of *OsIAA10* in rice leaves at different time point after RDV infection. The *OsEF1a* mRNA levels were used as internal controls. And then the value at time 0 was normalized to 1. Values are mean ± SD (n = 3 biological replicates). (B) Relative expression levels of *S2* in rice leaves at different time point after RDV infection. The *OsEF1a* mRNA levels were used as internal controls. And then the value at time 0 was normalized to 1. Values are mean ± SD (n = 3 biological replicates).
(TIF)

S6 Fig Construction of OsIAA10P116L-overexpressing transgenic rice. (A) Scheme showing domain II mutation in OsIAA10. Pro at position 116 of OsIAA10 was mutated into Leu by site-directed mutagenesis to yield OsIAA10P116L. (B) OsIAA10P116L does not interact with OsTIR1. Yeast two-hybrid assays were used to test interaction of OsIAA10P116L with OsTIR1 or with P2 on the SD-L-W-H-Ade medium in the presence or absence of IAA. (C) Cell-free degradation assays of MBP-OsIAA10 and MBP-OsIAA10P116L in rice cell-free extracts. Samples were collected at 0, 5, 15, 30, and 60 min after incubation at room temperature. Rubisco Large protein (RuL) was used as a loading control for total plant proteins. RL: relative level of remaining MBP-OsIAA10 protein. (D) Characterization and morphological phenotype of WT as well as OsIAA10- and OsIAA10P116L-overexpressing transgenic rice lines, respectively, at the seedling stage. Scale bars: 10cm.
(TIF)

S7 Fig Phenotypes of OsIAA10-overexpressing transgenic rice plants. (A) Morphology of OsIAA10-overexpressing transgenic rice plants at maturity stage. Scale bar: 15 cm. (B) Quantitative measurements of the tiller number, plant height, total grain number per panicle and rate of avoltive grain (%) for the plants in (A). The average (± SD) values were obtained from three biological repeats, with 15 plants from each line in each repeat. Significant differences (*P<0.05, **P< 0.01, n.s., no significant difference) are indicated based on Student’s *t*-test.
(TIF)

S8 Fig Auxin-mediated inhibition of root elongation is attenuated in OsIAA10-P116L-overexpressing rice plants. Root phenotypes of OsIAA10P116L- overexpressing rice lines after auxin treatment. Seeds of WT and M7 plants were germinated and grown in a liquid nutrient solution containing 0 or 0.1 μM NAA for 7 days before root length measurement. The left panel shows root phenotypes with or without 0.1 μM NAA. Scale bar, 0.5 cm. The right panel shows lengths of seminal roots of seedlings with or without 0.1 μM NAA. The average (± standard deviation) values were obtained from three biological repeats, with 15 plants from each line in each repeat. Significant differences (*P<0.05, **P< 0.01, ***P< 0.001) are indicated based on Student’s *t*-test.
(TIF)

S9 Fig RDV infection and OsIAA10P116L-overexpression reprogram auxin response in rice. qPCR analysis of auxin-induced gene expression after IAA treatment in M7 as well as Mock-inoculated and RDV-infected WT rice seedlings. The expression levels were normalized using the signal from *OsEF1a*, and values are mean ± SD (n = 3 biological replicates).
(TIF)

S10 Fig Characterization of OsIAA10 RNAi lines. (A) qPCR tests of *OsIAA10* expression in T2 generation of IAA10 RNAi lines (Ii-1-2 and Ii-10-1). *OsEF1a* was used as a reference. The average (± SD) values were obtained from three biological repeats. Significant differences (***P< 0.01) are indicated based on Student’s *t*-test. (B) Phenotype of OsIAA10 RNAi lines Ii-1-2, Ii-10-1 at seedling stage. Scale bar, 10 cm. Ii-1-2 and Ii-10-1 are two independent lines.
(TIF)

S11 Fig P2 does not alter OsIAA10 localization in tobacco leaves. GFP-OsIAA10 was expressed transiently with vector or HA-P2 in tobacco leaves for 3 days. Then the subcellular localizations of OsIAA10 in different leaves were observed under confocal microscopy. Nucleus marker SV40T-mCherry was used for co-localization analysis. Bar: 20 μm. On the right was western analysis for the protein expression of the samples for subcellular localization observing on the left.
(TIF)

S12 Fig Both the conserved domain and the left flanking domain of OsIAA10 domain II were essential for its interaction with P2. OsIAA10 DII, indicates OsIAA10 domain II. OsIAA10 DIII-IV, indicates OsIAA10 domain III-IV. OsIAA10 DIIΔC indicates OsIAA10 Domain II without the middle conserved domain. OsIAA10 DII-LC indicates OsIAA10 Domain II without the right flanking domain. OsIAA10 DII-CR indicates OsIAA10 Domain II without the left flanking domain. The OsIAA10 domain II amino acid sequences containing of the different domains were shown on the upper panel.
(TIF)

S13 Fig RDV infection affects both GA and Auxin pathway to cause dwarf symptom. (A) Infection of *d35* by RDV caused even more severe stunting of this rice mutant. Mock, mock inoculated; RDV, RDV infected. Bar: 10cm. (B). Schematic representation of plant height for the plants in (A). The average (± SD) values were obtained from three biological repeats, with 6 plants from each line in every repeat. Different letters indicate significant differences (*P*< 0.05) based on the Tukey-Kramer HSD test.
(TIF)

S14 Fig qPCR expression analysis of some selected genes that in SA and JA plant defense pathway. *OsEF1a* was used as a reference. The average (± SD) values were obtained from three biological repeats. Significant differences (*P< 0.05) are indicated based on Student’s *t*-test.
(TIF)

S1 Table Non-preference test for rice varieties and transgenic lines used in this study. (DOCX)

S2 Table Record of the number of rice lines showing RDV infection symptoms at time course for WT, L12, L20, M7 and M9. (DOCX)

S3 Table Record of the number of rice lines showing RDV infection symptoms at time course for WT, Ii-1-1 and Ii-10-1. (DOCX)

S4 Table Constructs list. (DOCX)

S5 Table Primers for plasmids constructions. (DOCX)

S6 Table Primers for Northern blot probes, qPCR, and RT-PCR. (DOCX)
