# Supplementary figures and images for: Rice Dwarf Virus P2 Protein Hijacks Auxin Signaling by Directly Targeting the Rice OsIAA10 Protein, Enhancing Viral Infection and Disease Development
Source: PLoS Pathog. 2016 Sep 8;12(9):e1005847. doi: 10.1371/journal.ppat.1005847 (PMC5015840; doi:10.1371/journal.ppat.1005847)

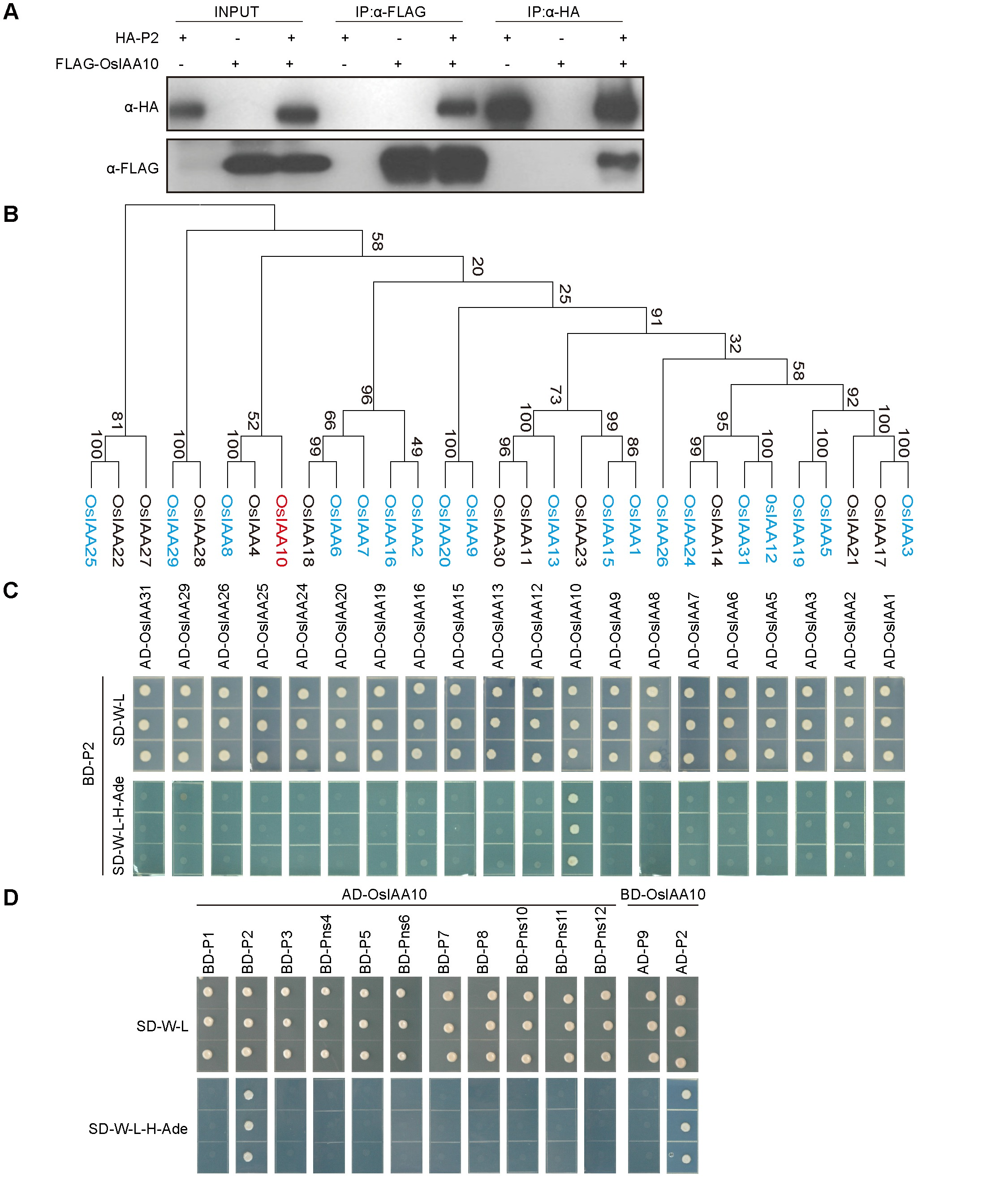

Supplement: S1 Fig — (A) Co-immunoprecipitation assays showing P2-OsIAA10 interaction in N. benthamiana leaves. (B) Phylogenetic relationship among the rice Aux/IAA proteins. The unrooted tree was generated using ClustalX program by neighbor-joining method. Bootstrap values form 100 replicates are indicated at each node. (C) Among the 20 rice Aux/IAA proteins tested, only OsIAA10 interacts with P2 in yeast two-hybrid assays. Yeast transformants were spotted on the control medium (SD-L-W) and selection medium (SD-L-W-H-Ade). (D) OsIAA10 specifically interacts with P2, but not other RDV proteins. (TIF) [file ppat.1005847.s001.tif]

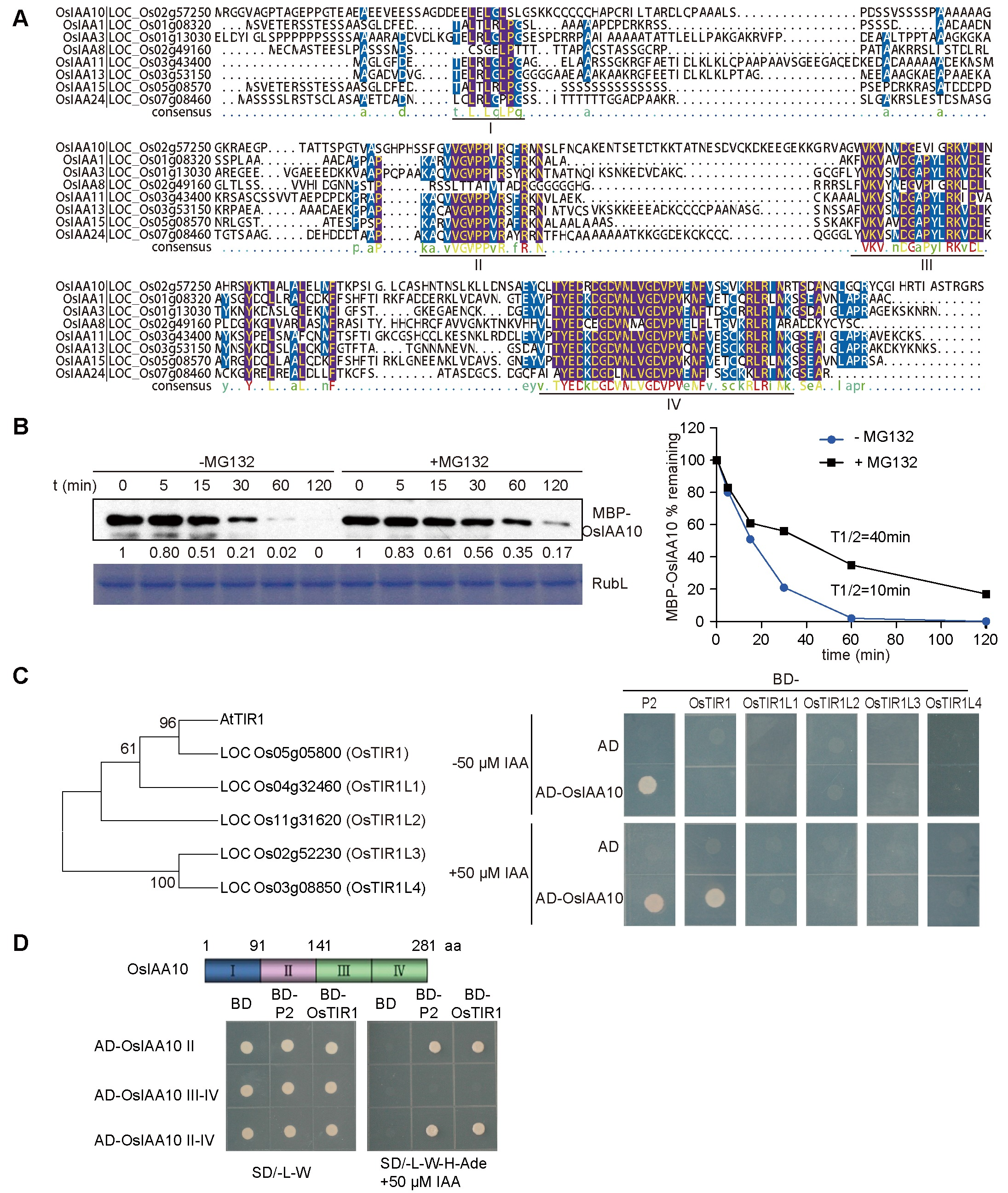

Supplement: S2 Fig — (A) OsIAA10 contains the four conserved domains (underlined), shared with the other Aux/IAA family members. (B) Cell-free degradation assays of MBP-OsIAA10 in the presence or absence of MG132 in rice extracts. +MG132: final MG132 concentration of 50 μM; -MG132: equal volume of DMSO (MG132 solvent) as a negative control. Samples were collected at 0, 5, 15, 30, 60, and 120 min after incubation at room temperature. Rubisco Large protein (RuL) was used as a loading control for total plant proteins. RL: relative level of remaining MBP-OsIAA10 protein. On the right are normalized plots for the degradation data of MBP-OsIAA10 shown on the left. (C) OsIAA10 interacts with OsTIR1 in the presence of auxin. Phylogenetic analysis of rice TIR1-like proteins is shown on the left. The unrooted tree was generated using ClustalX program by neighbor-joining method. Bootstrap values (above 50%) from 100 replicates are indicated at each node. The right panels show interaction or no interaction of OsIAA10 with OsTIR1 and other OsTIR1-like proteins in yeast. Yeast transformants were spotted on the selection medium (SD-L-W-H-Ade) with or without 50 μM IAA. (D) OsIAA10 domain II interacts with OsTIR1 in yeast. (TIF) [file ppat.1005847.s002.tif]

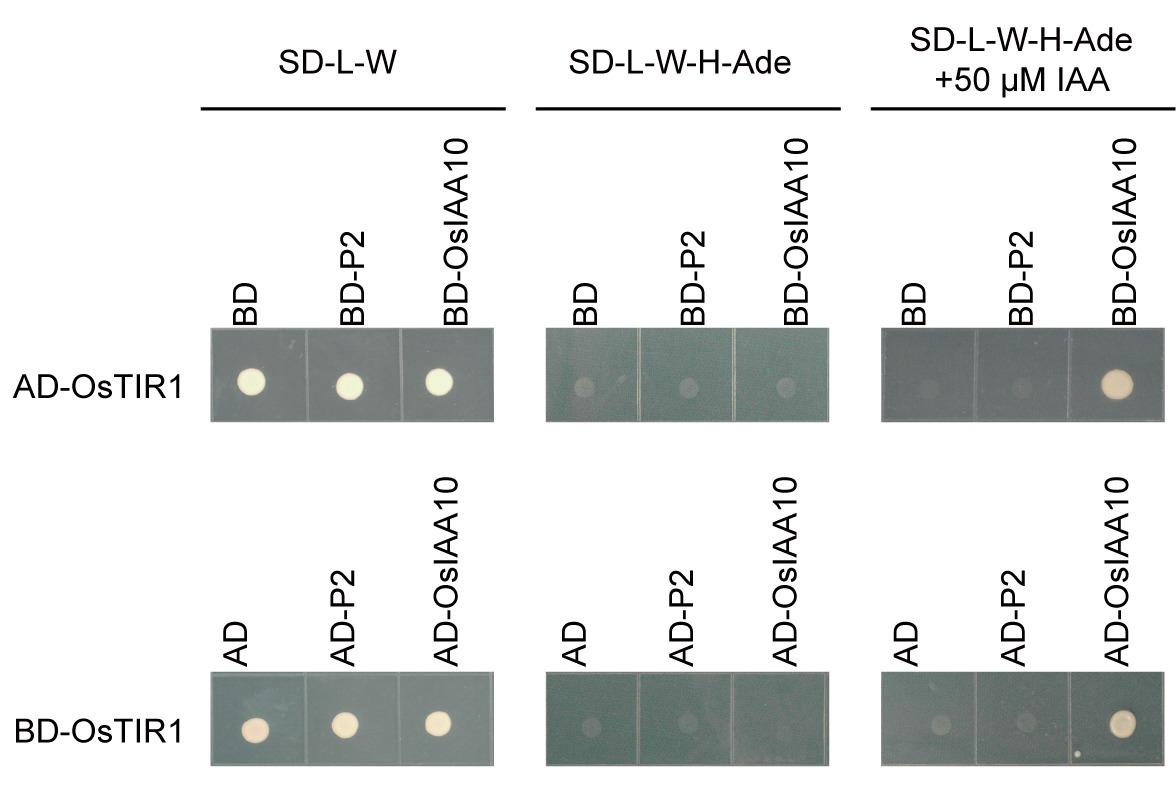

Supplement: S3 Fig — Yeast two-hybrid assays show that OsTIR1 does not interact with P2. Yeast transformants were spotted on the control medium (SD-L-W) and selection medium (SD-L-W-H-Ade) with or without 50 μM IAA. (TIF) [file ppat.1005847.s003.tif]

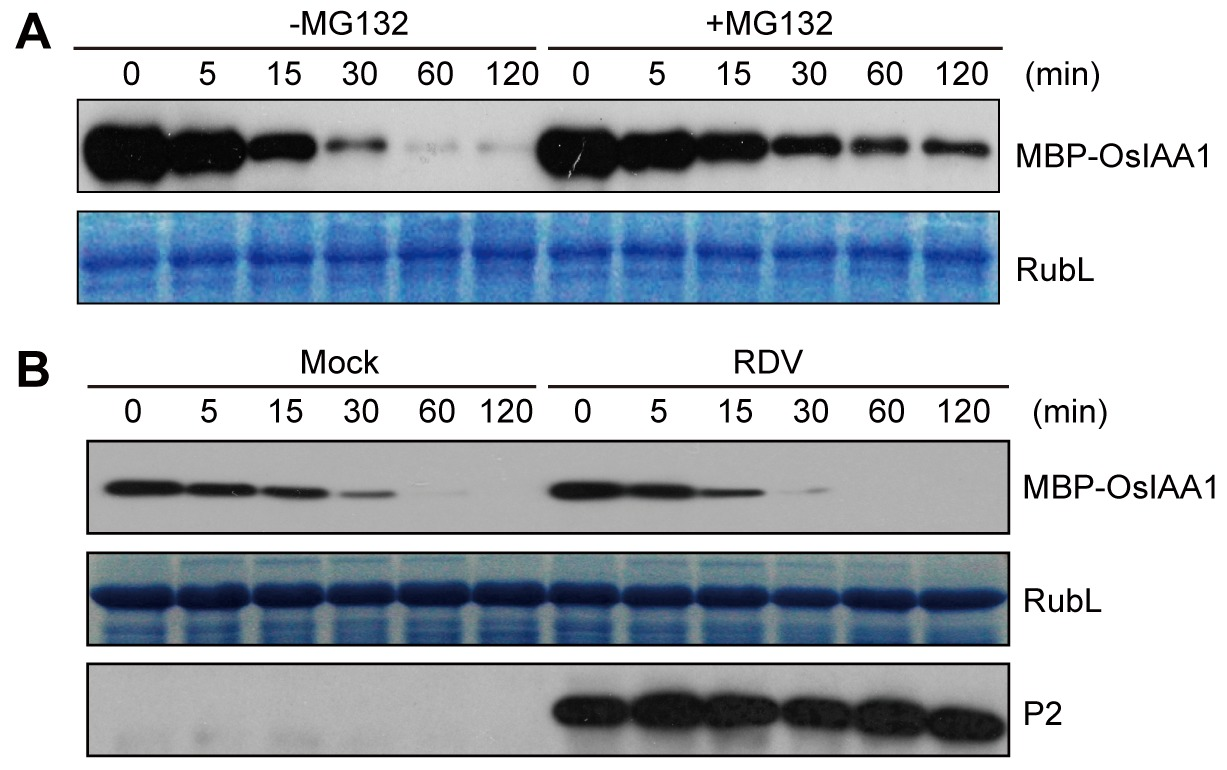

Supplement: S4 Fig — (A) Degradation of OsIAA1 is 26S proteasome-dependent. Cell-free degradation assays of MBP-OsIAA1 in the presence or absence of MG132 in rice extracts. +MG132, final MG132 concentration of 50 μM; -MG132, equal volume of DMSO (MG132 solvent) as a negative control. (B) Cell-free degradation assays of MBP-OsIAA1 in mock or RDV-infected rice extracts. Samples were collected at 0, 5, 15, 30, 60 and 120 min after incubation at room temperature. Rubisco Large protein (RuL) was used as a loading control of total plant protein. RL: relative level of remaining MBP-OsIAA1 protein. (TIF) [file ppat.1005847.s004.tif]

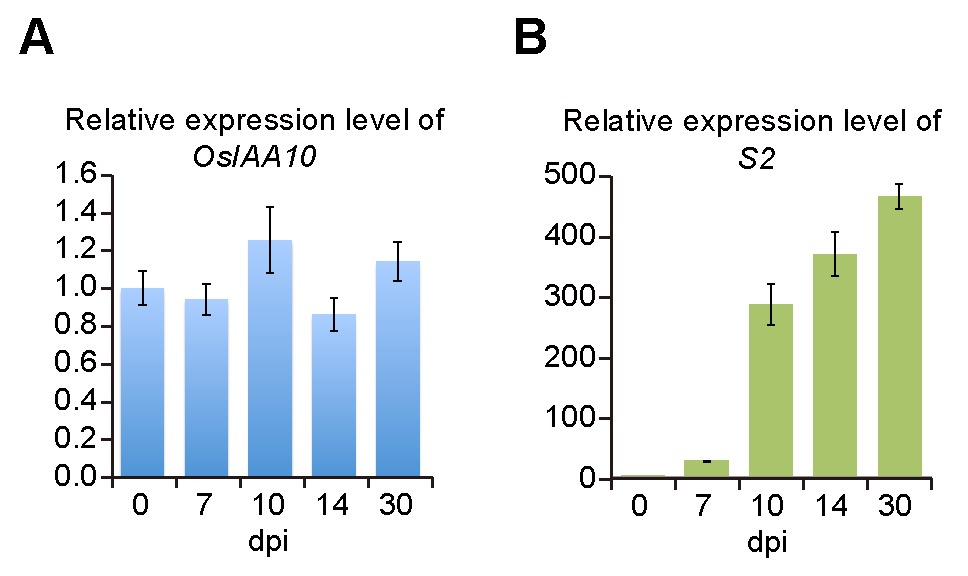

Supplement: S5 Fig — (A) Relative expression levels of OsIAA10 in rice leaves at different time point after RDV infection. The OsEF1a mRNA levels were used as internal controls. And then the value at time 0 was normalized to 1. Values are mean ± SD (n = 3 biological replicates). (B) Relative expression levels of S2 in rice leaves at different time point after RDV infection. The OsEF1a mRNA levels were used as internal controls. And then the value at time 0 was normalized to 1. Values are mean ± SD (n = 3 biological replicates). (TIF) [file ppat.1005847.s005.tif]

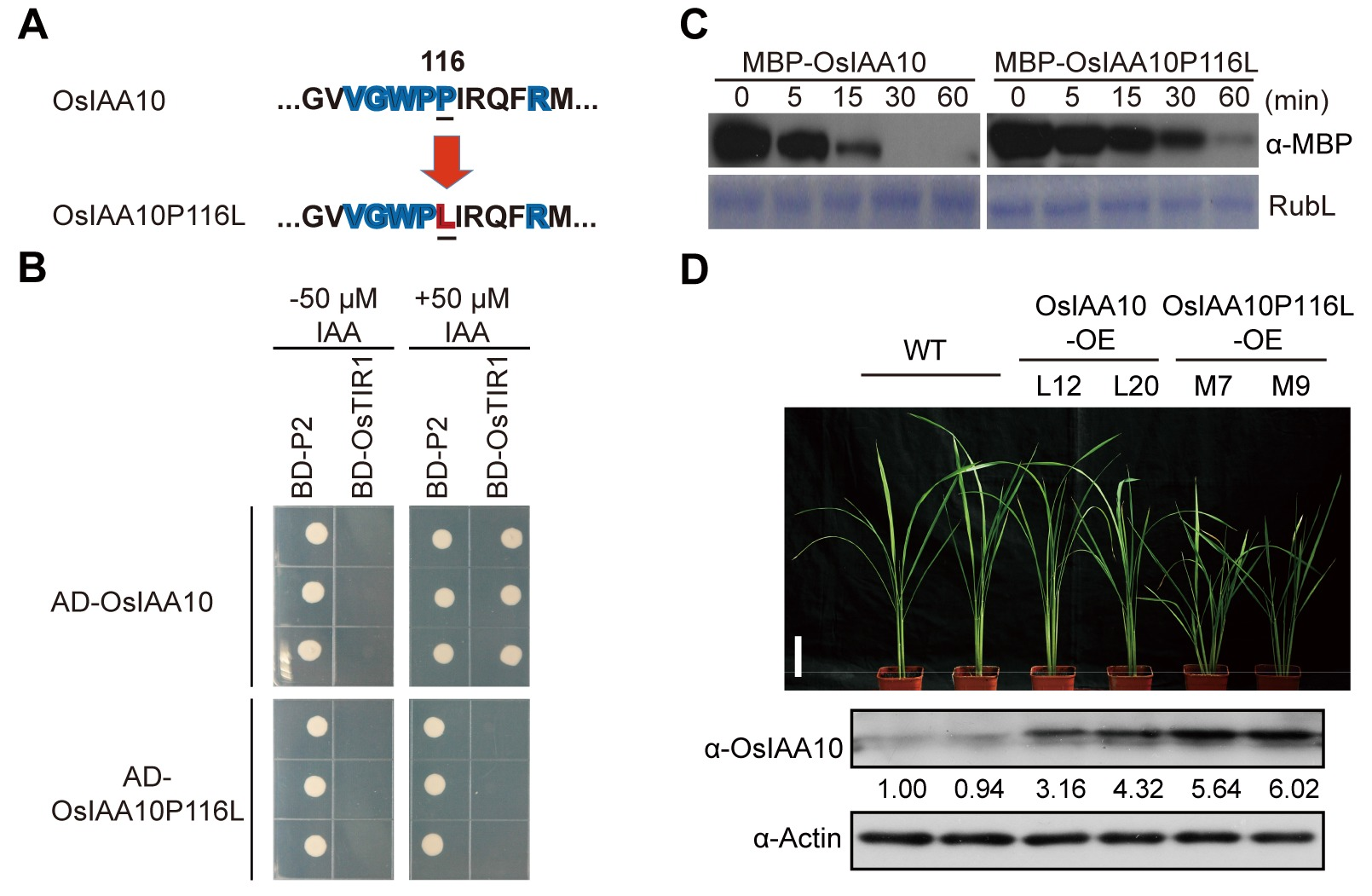

Supplement: S6 Fig — (A) Scheme showing domain II mutation in OsIAA10. Pro at position 116 of OsIAA10 was mutated into Leu by site-directed mutagenesis to yield OsIAA10P116L. (B) OsIAA10P116L does not interact with OsTIR1. Yeast two-hybrid assays were used to test interaction of OsIAA10P116L with OsTIR1 or with P2 on the SD-L-W-H-Ade medium in the presence or absence of IAA. (C) Cell-free degradation assays of MBP-OsIAA10 and MBP-OsIAA10P116L in rice cell-free extracts. Samples were collected at 0, 5, 15, 30, and 60 min after incubation at room temperature. Rubisco Large protein (RuL) was used as a loading control for total plant proteins. RL: relative level of remaining MBP-OsIAA10 protein. (D) Characterization and morphological phenotype of WT as well as OsIAA10- and OsIAA10P116L-overexpressing transgenic rice lines, respectively, at the seedling stage. Scale bars: 10cm. (TIF) [file ppat.1005847.s006.tif]

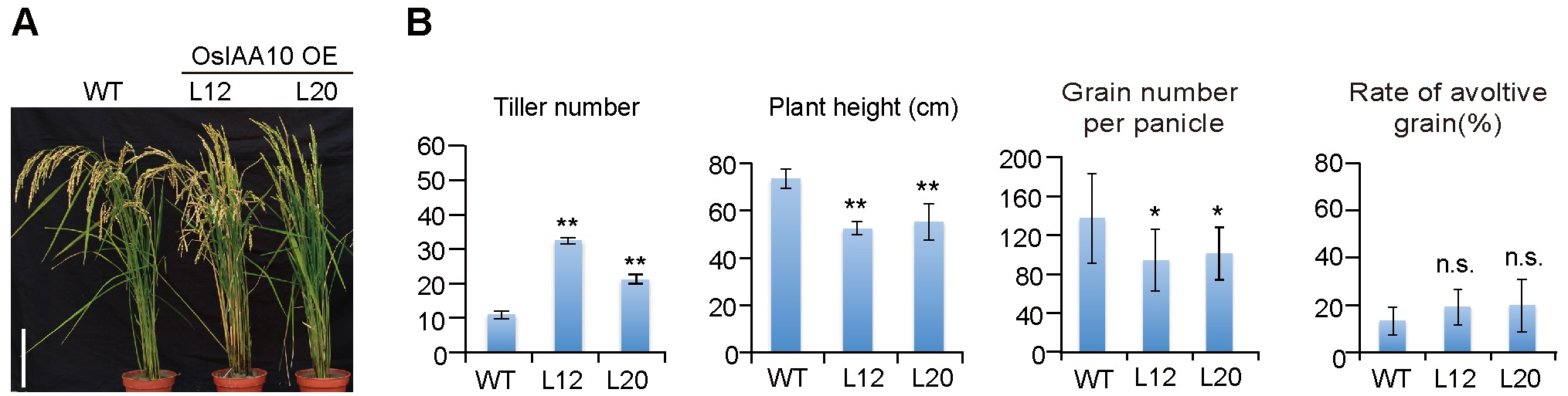

Supplement: S7 Fig — (A) Morphology of OsIAA10-overexpressing transgenic rice plants at maturity stage. Scale bar: 15 cm. (B) Quantitative measurements of the tiller number, plant height, total grain number per panicle and rate of avoltive grain (%) for the plants in (A). The average (± SD) values were obtained from three biological repeats, with 15 plants from each line in each repeat. Significant differences (*P<0.05, **P< 0.01, n.s., no significant difference) are indicated based on Student’s t-test. (TIF) [file ppat.1005847.s007.tif]

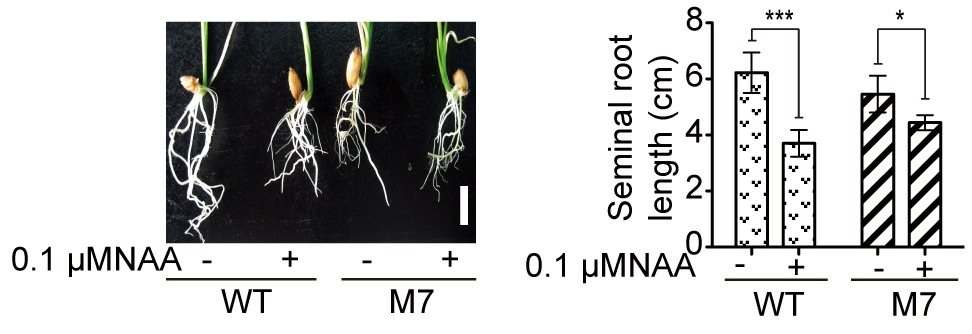

Supplement: S8 Fig — Root phenotypes of OsIAA10P116L- overexpressing rice lines after auxin treatment. Seeds of WT and M7 plants were germinated and grown in a liquid nutrient solution containing 0 or 0.1 μM NAA for 7 days before root length measurement. The left panel shows root phenotypes with or without 0.1 μM NAA. Scale bar, 0.5 cm. The right panel shows lengths of seminal roots of seedlings with or without 0.1 μM NAA. The average (± standard deviation) values were obtained from three biological repeats, with 15 plants from each line in each repeat. Significant differences (*P<0.05, **P< 0.01, ***P< 0.001) are indicated based on Student’s t-test. (TIF) [file ppat.1005847.s008.tif]

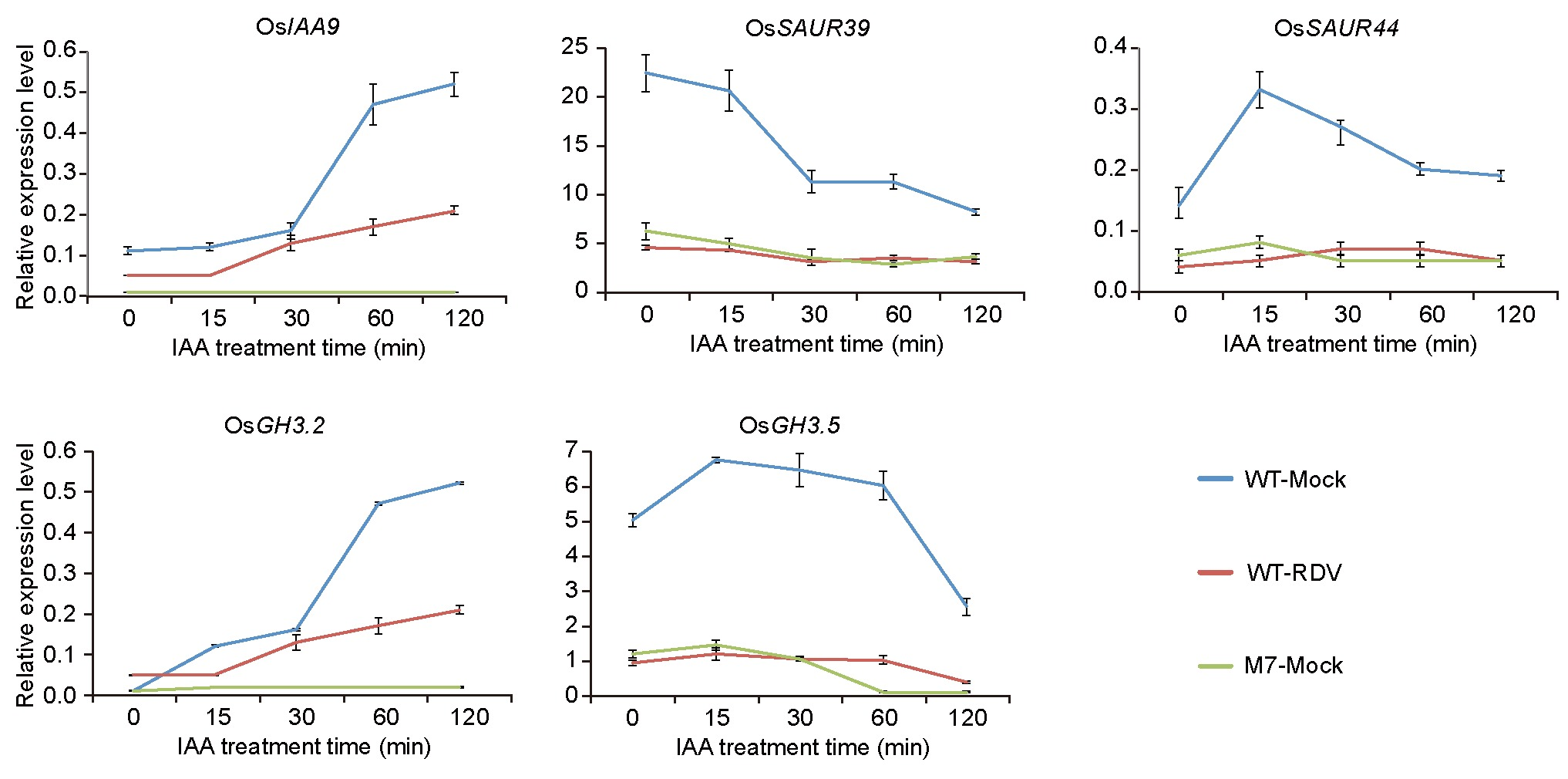

Supplement: S9 Fig — qPCR analysis of auxin-induced gene expression after IAA treatment in M7 as well as Mock-inoculated and RDV-infected WT rice seedlings. The expression levels were normalized using the signal from OsEF1a, and values are mean ± SD (n = 3 biological replicates). (TIF) [file ppat.1005847.s009.tif]

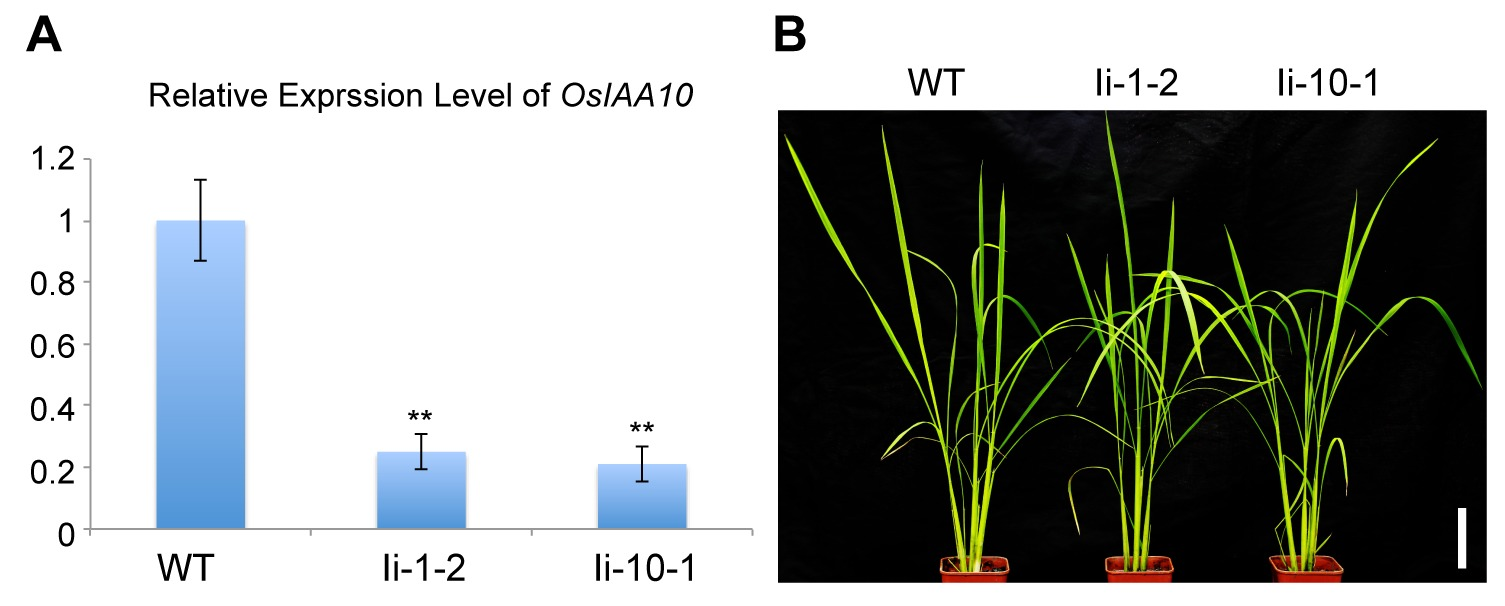

Supplement: S10 Fig — (A) qPCR tests of OsIAA10 expression in T2 generation of IAA10 RNAi lines (Ii-1-2 and Ii-10-1). OsEF1a was used as a reference. The average (± SD) values were obtained from three biological repeats. Significant differences (***P< 0.01) are indicated based on Student’s t-test. (B) Phenotype of OsIAA10 RNAi lines Ii-1-2, Ii-10-1 at seedling stage. Scale bar, 10 cm. Ii-1-2 and Ii-10-1 are two independent lines. (TIF) [file ppat.1005847.s010.tif]

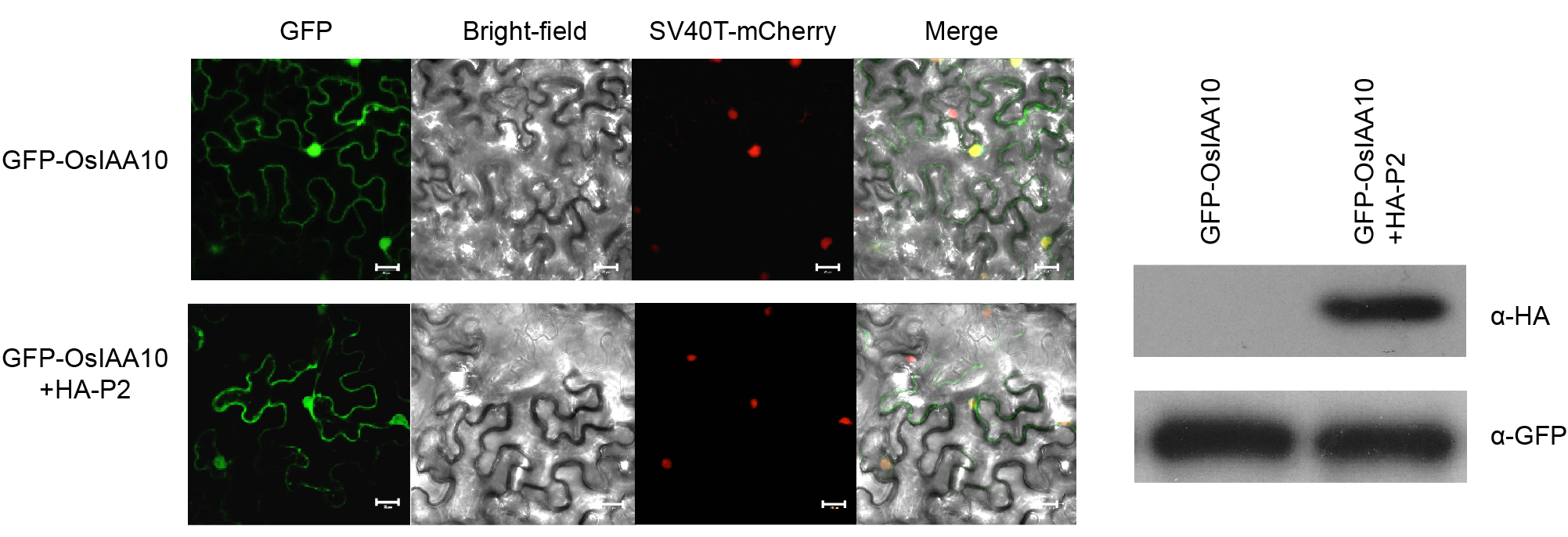

Supplement: S11 Fig — GFP-OsIAA10 was expressed transiently with vector or HA-P2 in tobacco leaves for 3 days. Then the subcellular localizations of OsIAA10 in different leaves were observed under confocal microscopy. Nucleus marker SV40T-mCherry was used for co-localization analysis. Bar: 20 μm. On the right was western analysis for the protein expression of the samples for subcellular localization observing on the left. (TIF) [file ppat.1005847.s011.tif]

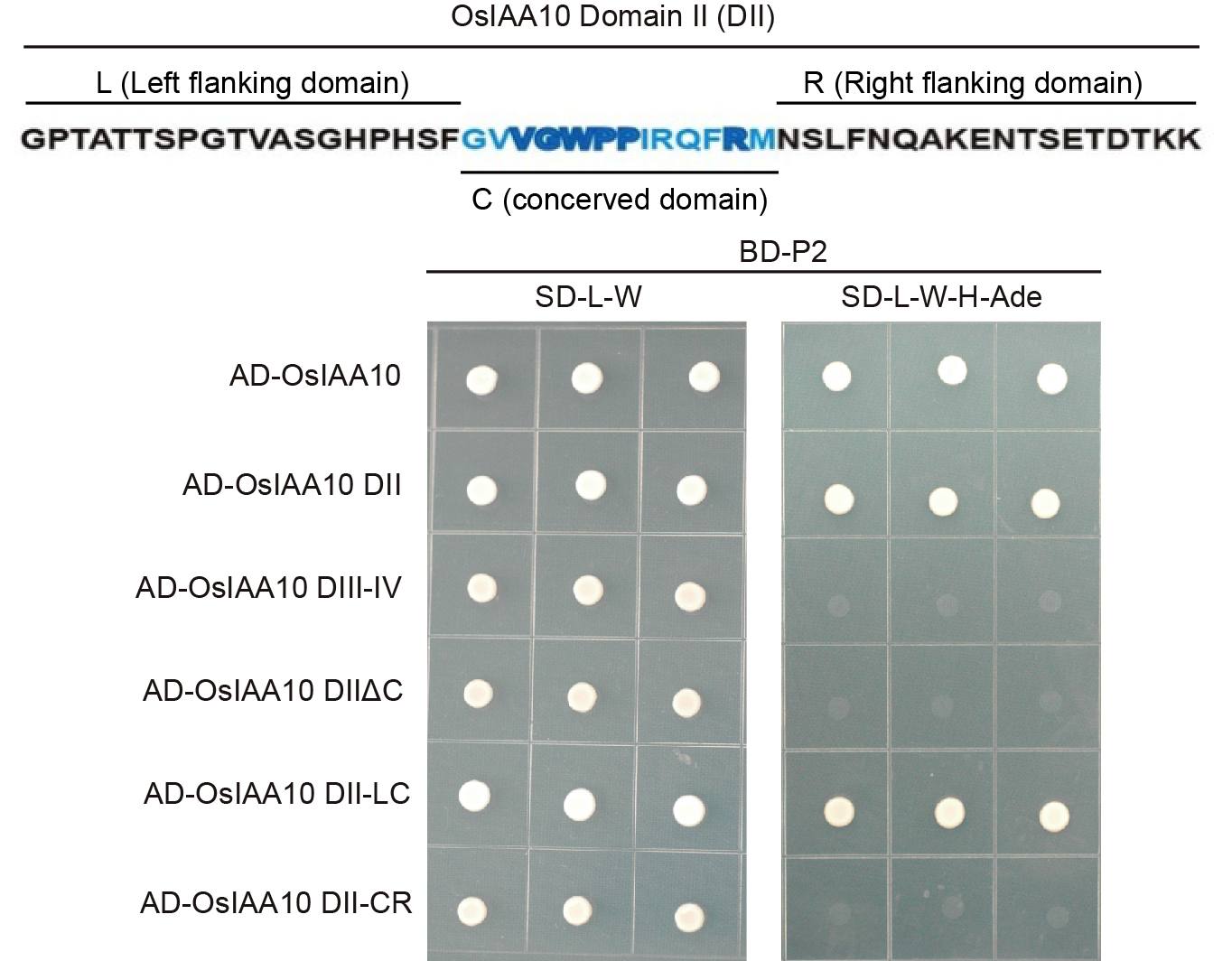

Supplement: S12 Fig — OsIAA10 DII, indicates OsIAA10 domain II. OsIAA10 DIII-IV, indicates OsIAA10 domain III-IV. OsIAA10 DIIΔC indicates OsIAA10 Domain II without the middle conserved domain. OsIAA10 DII-LC indicates OsIAA10 Domain II without the right flanking domain. OsIAA10 DII-CR indicates OsIAA10 Domain II without the left flanking domain. The OsIAA10 domain II amino acid sequences containing of the different domains were shown on the upper panel. (TIF) [file ppat.1005847.s012.tif]

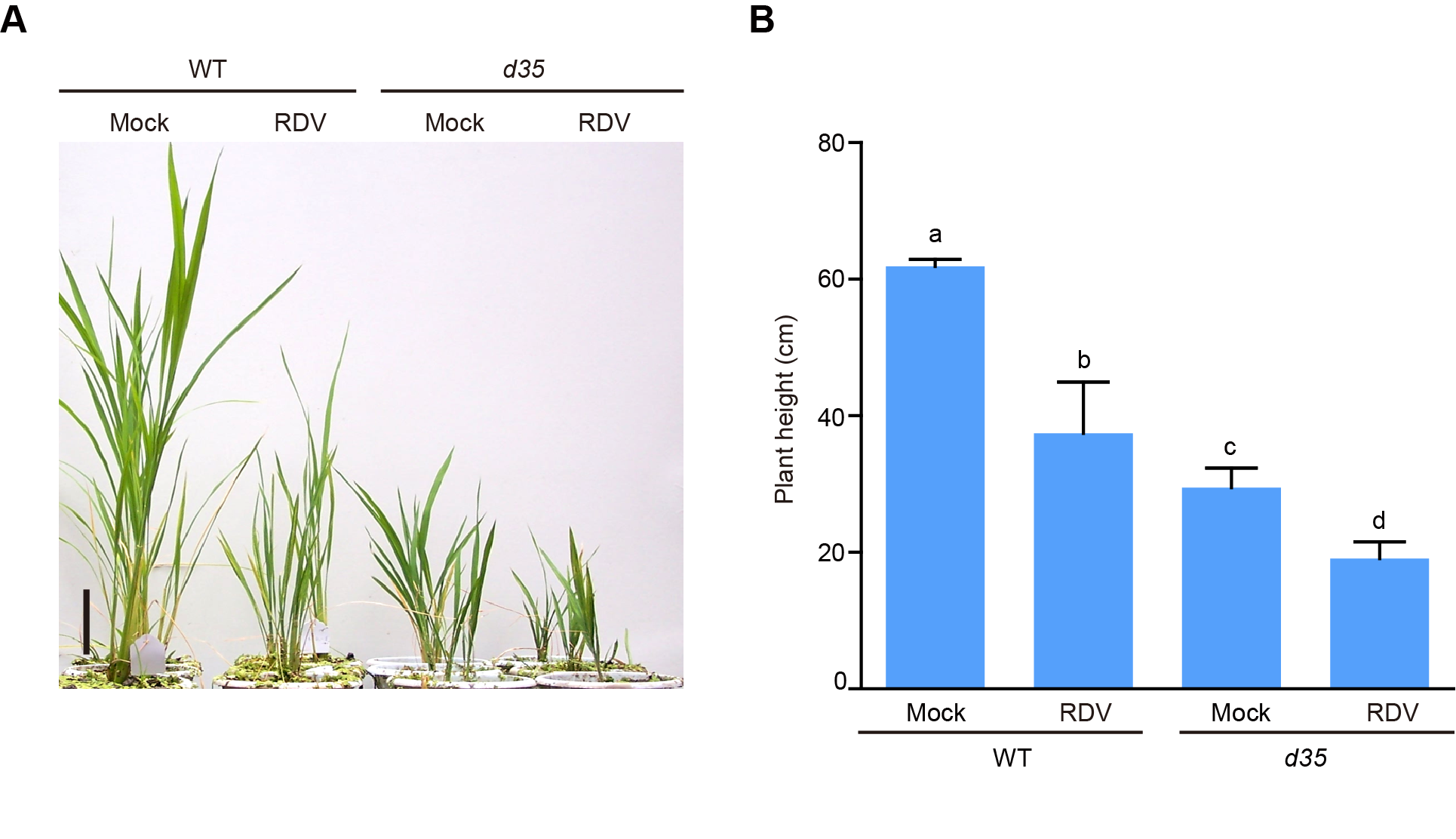

Supplement: S13 Fig — (A) Infection of d35 by RDV caused even more severe stunting of this rice mutant. Mock, mock inoculated; RDV, RDV infected. Bar: 10cm. (B). Schematic representation of plant height for the plants in (A). The average (± SD) values were obtained from three biological repeats, with 6 plants from each line in every repeat. Different letters indicate significant differences (P< 0.05) based on the Tukey-Kramer HSD test. (TIF) [file ppat.1005847.s013.tif]

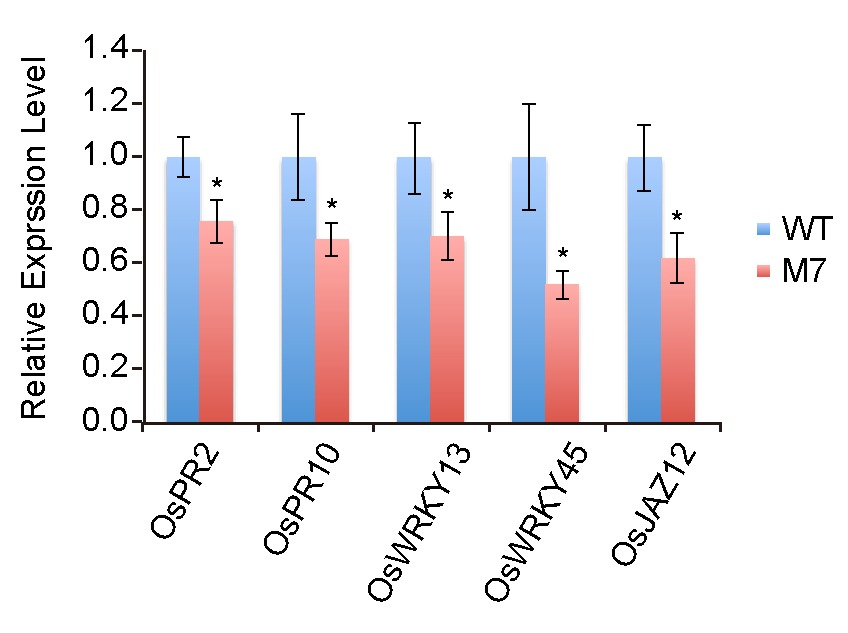

Supplement: S14 Fig — OsEF1a was used as a reference. The average (± SD) values were obtained from three biological repeats. Significant differences (*P< 0.05) are indicated based on Student’s t-test. (TIF) [file ppat.1005847.s014.tif]
