# Supplementary material for: Rice Dwarf Virus P2 Protein Hijacks Auxin Signaling by Directly Targeting the Rice OsIAA10 Protein, Enhancing Viral Infection and Disease Development
Source: PLoS Pathog. 2016 Sep 8;12(9):e1005847. doi: 10.1371/journal.ppat.1005847 (PMC5015840; doi:10.1371/journal.ppat.1005847)
Supplement: S1 Table — (DOCX) [file ppat.1005847.s015.docx]

**S1 Table. Non-preference test for rice varieties and transgenic lines used in this study.**

| **Varieties** | **Non-preference^*1^** |
| --- | --- |
| ZH11 | 1.95^a*2^ |
| L12 | 2.15^a^ |
| L20 | 2.05^a^ |
| M7 | 1.85^a^ |
| M9 | 1.75^a^ |
| Ii-1-2 | 2.15^a^ |
| Ii-10-1 | 1.85^a^ |

*1, Non-preference was indicated by the number of leafhoppers settled on the individual plant.

*2, “a” means there is no significant difference (P value> 0.05) between these data.
