# Supplementary material for: Rice Dwarf Virus P2 Protein Hijacks Auxin Signaling by Directly Targeting the Rice OsIAA10 Protein, Enhancing Viral Infection and Disease Development
Source: PLoS Pathog. 2016 Sep 8;12(9):e1005847. doi: 10.1371/journal.ppat.1005847 (PMC5015840; doi:10.1371/journal.ppat.1005847)
Supplement: S2 Table — (DOCX) [file ppat.1005847.s016.docx]

**S3 Table. Record of the number of rice lines showing RDV infection symptoms at time course for WT, L12, L20, M7 and M9^*1^ plants.**

|  |  | WT  (Total: 20/repeat)^*2^ | L12  (Total: 20/repeat) | L20  (Total: 20/repeat) | M7  (Total: 20/repeat) | M9  (Total: 20/repeat) |
| --- | --- | --- | --- | --- | --- | --- |
| 1wpi^*3^ | R1^*4^ | 0 | 3 | 0 | 2 | 2 |
|  | R2 | 1 | 3 | 1 | 3 | 3 |
|  | R3 | 0 | 4 | 1 | 5 | 4 |
| 2wpi | R1 | 2 | 6 | 3 | 9 | 7 |
|  | R2 | 2 | 7 | 4 | 11 | 8 |
|  | R3 | 2 | 6 | 3 | 10 | 8 |
| 3wpi | R1 | 6 | 12 | 8 | 12 | 12 |
|  | R2 | 7 | 12 | 10 | 15 | 14 |
|  | R3 | 5 | 11 | 9 | 13 | 12 |
| 4wpi | R1 | 10 | 15 | 12 | 18 | 19 |
|  | R2 | 11 | 16 | 12 | 19 | 19 |
|  | R3 | 10 | 15 | 12 | 18 | 19 |
| 5wpi | R1 | 11 | 16 | 13 | 19 | 19 |
|  | R2 | 12 | 17 | 14 | 19 | 20 |
|  | R3 | 11 | 16 | 15 | 18 | 19 |
| 6wpi | R1 | 15 | 18 | 17 | 20 | 20 |
|  | R2 | 14 | 17 | 17 | 19 | 20 |
|  | R3 | 15 | 18 | 17 | 20 | 20 |
| 7wpi | R1 | 17 | 18 | 18 | 20 | 20 |
|  | R2 | 16 | 18 | 17 | 20 | 20 |
|  | R3 | 16 | 19 | 18 | 20 | 20 |
| 8wpi | R1 | 17 | 19 | 18 | 20 | 20 |
|  | R2 | 17 | 19 | 19 | 20 | 20 |
|  | R3 | 17 | 20 | 18 | 20 | 20 |

*1: WT, wild type rice; L12 and L20, two independent OsIAA10 overexpression rice lines; M7 and M9, two independent OsIAA10P116L overexpression rice lines.

*2: For each repeat, 20 seedlings were inoculated with viruliferous leafhopper.

*3: 1wpi means 1 week-post-inoculation.

*4: R1 means biological Repeat 1.
