# Supplementary material for: Rice Dwarf Virus P2 Protein Hijacks Auxin Signaling by Directly Targeting the Rice OsIAA10 Protein, Enhancing Viral Infection and Disease Development
Source: PLoS Pathog. 2016 Sep 8;12(9):e1005847. doi: 10.1371/journal.ppat.1005847 (PMC5015840; doi:10.1371/journal.ppat.1005847)
Supplement: S3 Table — (DOCX) [file ppat.1005847.s017.docx]

**S4 Table. Record of the number of rice lines showing RDV infection symptoms at time course for WT, Ii-1-1 and Ii-10-1^*1^ plants.**

|  |  | WT  (Total: 20/repeat) ^*2^ | Ii-1-1  (Total: 20/repeat) | Ii-10-1  (Total: 20/repeat) |
| --- | --- | --- | --- | --- |
| 1wpi^*3^ | R1^*4^ | 1 | 0 | 0 |
|  | R2 | 1 | 1 | 0 |
|  | R3 | 0 | 0 | 0 |
| 2wpi | R1 | 4 | 1 | 2 |
|  | R2 | 5 | 2 | 3 |
|  | R3 | 2 | 0 | 1 |
| 3wpi | R1 | 8 | 2 | 3 |
|  | R2 | 7 | 3 | 4 |
|  | R3 | 6 | 2 | 3 |
| 4wpi | R1 | 9 | 4 | 5 |
|  | R2 | 11 | 5 | 5 |
|  | R3 | 10 | 3 | 4 |
| 5wpi | R1 | 10 | 7 | 7 |
|  | R2 | 12 | 7 | 9 |
|  | R3 | 11 | 6 | 8 |
| 6wpi | R1 | 17 | 8 | 11 |
|  | R2 | 14 | 9 | 10 |
|  | R3 | 15 | 7 | 10 |
| 7wpi | R1 | 17 | 11 | 14 |
|  | R2 | 16 | 12 | 13 |
|  | R3 | 17 | 11 | 13 |
| 8wpi | R1 | 18 | 11 | 14 |
|  | R2 | 17 | 12 | 13 |
|  | R3 | 17 | 11 | 13 |

*1: WT, wild type rice; Ii-1-2 and Ii-10-1, two independent OsIAA10RNAi transgenic rice lines.

*2: For each repeat, 20 seedlings were inoculated with viruliferous leafhopper.

*3: 1wpi means 1 week-post-inoculation.

*4: R1 means biological Repeat 1.
