# Supplementary material for: Rice Dwarf Virus P2 Protein Hijacks Auxin Signaling by Directly Targeting the Rice OsIAA10 Protein, Enhancing Viral Infection and Disease Development
Source: PLoS Pathog. 2016 Sep 8;12(9):e1005847. doi: 10.1371/journal.ppat.1005847 (PMC5015840; doi:10.1371/journal.ppat.1005847)
Supplement: S4 Table — (DOCX) [file ppat.1005847.s018.docx]

**S4 Table. Constructs list.**

| **use** | **Construct** | **Plasmid name** | **Insert Fragment** | **Primers** | **Template** | **Plasmid backbone** | **Cloning method** |
| --- | --- | --- | --- | --- | --- | --- | --- |
| Yeast  Two-  hybrid | AD-P2 | pGAD-S2 | S2 | S2F1  S2R1 | RDV-infected rice cDNA | pGAD-T7 | *Eco*R I  *Bam*H I |
|  | BD-P2 | pGBK-S2 | S2 | S2F1  S2R1 | pGAD-S2 | pGBK-T7 | *Eco*R I  *Bam*H I |
|  | BD-P2 (1-786) | pGBK-  S2(1-786) | S2 (1-2358) | S2F1  S2(2358)R1 | pGBK-S2 | pGBK-T7 | *Eco*R I  *Bam*H I |
|  | BD-P2 (1-140) | pGBK-  S2(1-140) | S2 (1-420) | S2F1  S2(420)R | pGBK-S2 | pGBK-T7 | *Eco*R I  *Bam*H I |
|  | BD-P2 (Δ1-80) | pGBK-  S2(Δ1-80) | S2 (Δ1-240) | S2(241)F  S2R1 | pGBK-S2 | pGBK-T7 | *Eco*R I  *Bam*H I |
|  | BD-P2 (91-140) | pGBK-  S2(91-140) | S2 (271-420) | S2(271)F  S2(420)R | pGBK-S2 | pGBK-T7 | *Eco*R I  *Bam*H I |
|  | BD-P2 (49-90) | pGBK-  S2(49-90) | S2 (148-270) | S2(148)F  S2(270)R | pGBK-S2 | pGBKT7 | *Eco*R I  *Bam*H I |
|  | BD-P2 (1-50) | pGBK-  S2(1-50) | S2 (1-150) | S2F1  S2(150)R | pGBK-S2 | pGBK-T7 | *Eco*R I  *Bam*H I |
|  | AD-OsIAA10 | pGAD-  OsIAA10 | OsIAA10 | IAA10F1  IAA10R1 | Rice cDNA | pGAD-T7 | *Bam*H I  *Xho* I |
|  | AD-OSIAA10  P116L | pGAD-  OsIAA10m | OsIAA10 (C347T) | IAA10mF  IAA10mR | pGAD-  OsIAA10 | pGAD-T7 | KOD-Plus-Mutagenesis -Kit |
|  | AD-OSIAA10II | pGAD-  OsIAA10II | OsIAA10 (274-420) | IAA10(274)F  IAA10(420)R | pGAD-  OsIAA10 | pGAD-T7 | *Bam*H I  *Xho* I |
|  | AD-OSIAA10  III-IV | pGAD-OsIAA10III-IV | OsIAA10 (Δ1-420) | IAA10(421)F  IAA10R1 | pGAD-  OsIAA10 | pGAD-T7 | *Bam*H I  *Xho* I |
|  | BD-OsTIR1 | pGBK-  OsTIR1 | OsTIR1 | OsTIR1F1  OsTIR1R1 | Rice cDNA | pGBK-T7 | *Eco*R I  *Bam*H I |
|  | AD-OsTIR1 | pGAD-  OsTIR1 | OsTIR1 | OsTIR1F1  OsTIR1R1 | Rice cDNA | pGAD-T7 | *Eco*R I  *Bam*H I |
|  | BD-TIR1L1 | pGBK-  OsTIR1L1 | OsTIR1L1 | OsTIR1L1F  OsTIR1L1R | Rice cDNA | pGBK-T7 | *Eco*R I  *Bam*H I |
|  | BD-TIR1L2 | pGBK-  OsTIR1L2 | OsTIR1L2 | OsTIR1L2F  OsTIR1L2R | Rice cDNA | pGBK-T7 | *Bam*H I  *Sal* I |
|  | BD-TIR1L3 | pGBK-  OsTIR1L3 | OsTIR1L3 | OsTIR1L3F  OsTIR1L3R | Rice cDNA | pGBK-T7 | *Nde* I  *Bam*H I |
|  | BD-TIR1L4 | pGBk-  OsTIR1L4 | OsTIR1L4 | OsTIR1L4F  OsTIR1L4R | Rice cDNA | pGBK-T7 | *Eco*R I  *Bam*H I |
| Transgene rice and transient expression in tobacco | 35S:FLAG-  OsIAA10 | p1301-FLAG-OsIAA10 | FLAG  -OsIAA10 | FLAGIAA10F1  IAA10R2 | pGAD-  OsIAA10 | pCAMBIA1301 | *Sal* I  *Bam*H I |
|  | 35S:FLAG-Os  IAA10P116L | p1301-FLAG-OsIAA10m | FLAG-OsIAA10(C347T) | FLAGIAA10F1  IAA10R2 | pGAD-  OsIAA10m | pCAMBIA1301 | *Sal* I  *Bam*H I |
|  | 35S:HA-  OsTIR1 | pWM101-HAOsTIR1 | HAOsTIR1 | HAOsTIR1F1  OsTIR1R2 | pGBK-  OsTIR1 | pWM101 | *Kpn* I  *Sal*I |
| LCI assay | cLUC-S2 | p1300-cLUC-S2 | HAS2 | HAS2F1  S2R2 | pGBK-S2 | pCMABIA1300 | *Bam*H I  *Sal* I |
|  | OsIAA10-nLUC | p1300-nLUC-OsIAA10 | FLAG-  OsIAA10 | FLAGIAA10F2  IAA10R3 | pGAD-  OsIAA10 | pCMABIA1300 | *Kpn* I  *Sal* I |
| Rice transgene | Actin: IAA10RNAi | p2300-OsIAA10RNAi | OsIAA10 and OsIAA10IR | IAA10F2  IAA10R4 | pGAD-  OsIAA10 | pCAMBIA2300 | *Spe* I  *Bgl*I I |
| Protein puri  -fication for pulldown  and  MST assay | MBP-OSIAA10 | pMAL-p2x- OsIAA10 | OsIAA10 | IAA10F3  IAA10R5 | pGAD-  OsIAA10 | pMAL-p2x | *Sal* I  *Hind* III |
|  | MBP-OsIAA10P116L | pMAL-p2x- OsIAA10m | OsIAA10  (C347T) | IAA10F1  IAA10R6 | pGAD-  OsIAA10m | pMAL-p2x | *Bam*H I  *Sal* I |
|  | MBP-OsTIR1 | pMAL-p2x- OsTIR1 | OsTIR1 | OsTIR1F2  OsTIR1R2 | pGBK-  OsTIR1 | pMAL-p2x | *Bam*H I  *Sal*I |
|  | MBP-OsIAA1 | pMAL-p2x- OsIAA1 | OsIAA1 | OsIAA1F  OsIAA1R | Rice cDNA | pMAL-p2x | *Bam*H I  *Sal* I |
|  | MBP-P2 (1-786) | pMAL-His- P2 (1-786) | S2(1-2358) | S2F1  S2(2358)R1 | pGBK-S2 | pMAL-p2x | *Eco*R I  *Bam*H I |
|  | GST-P2 (1-786) | pCST-P2 (1-786) | S2(1-2358) | S2F1  S2(2358)R1 | pGBK-S2 | pCST-AXL II | *Eco*R I  *Bam*H I |
|  | GST-OsIAA10 | pGEX-4T-1- OsIAA10 | OsIAA10 | IAA10F1  IAA10R1 | pGAD-  OsIAA10 | pGEX-  4T-1 | *Bam*H I  *Xho* I |
