# Supplementary material for: Rice Dwarf Virus P2 Protein Hijacks Auxin Signaling by Directly Targeting the Rice OsIAA10 Protein, Enhancing Viral Infection and Disease Development
Source: PLoS Pathog. 2016 Sep 8;12(9):e1005847. doi: 10.1371/journal.ppat.1005847 (PMC5015840; doi:10.1371/journal.ppat.1005847)
Supplement: S5 Table — (DOCX) [file ppat.1005847.s019.docx]

**S5 Table. Primers for plasmids constructions.**

| **Primer name** | **Primer Sequence 5’-3’** |
| --- | --- |
| S2F1 | GCGAATTCATGGCTTATCCTAATGACGTCAG |
| S2(2358)R1 | GCGGATCCTCACTGCTGCGCACGTGTGG |
| S2(420)R | GCGGATCCTCACATAGACTGATCTCCTGT |
| S2R1 | GCGGATCCTCACAATGCATCATAGATAGATTGG |
| S2(270)R | GCGGATCCTCATAAGACTGCGGCACGGTCG |
| S2(150)R | GCGGATCCTCAATCGCGCTCCATATTAGTAAG |
| S2(241)F | GCGAATTCATGGATGCCGACCGTGCCGCAGT |
| HAS2F1 | GCGGATCCGATGTACCCATACGATGTTCCAGATTACGCGATGGCTTATCCTAATGACGTCAG |
| S2R2 | GAGTCGACTCACAATGCATCATAGATAGATTG |
| IAA10F1 | GTGGATCCAAATGAGAGGAGGAGTAGCTGG |
| IAA10R1 | GCCTCGAGTCAGGATCTGCCTCTTGTTG |
| IAA10mF | TGGGATGGCCACTGATCCGACAGTT |
| IAA10mR | AACTGTCGGATCAGTGGCCATCCCA |
| IAA10(274)F | AAGGATCCTACCCACCGCCACCACCTCGCCTG |
| IAA10(420)R | GCCTCGAGTTCTTGGTGTCGGTTTC |
| IAA10(421)F | AAGGATCCTAACCGCCACCAATGAATCTG |
| FLAGIAA10F1 | CCGTCGACATGGACTACAAGGACGACGATGACAAGATGAGAGGAGGAGTAGCTG |
| IAA10R2 | GCGGATCCTCAGGATCTGCCTCTTGTTG |
| FLAGIAA10F2 | CCGGTACCATGGACTACAAGGACGACGATGACAAGATGAGAGGAGGAGTAGCTG |
| IAA10R3 | GCGTCGACGGATCTGCCTCTTGTTG |
| IAA10F2 | GTACTAGTATGAGAGGAGGAGTAGCTGG |
| IAA10R4 | GCAGATCTTCAGGATCTGCCTCTTGTTG |
| IAA10F3 | GTGTCGACATGAGAGGAGGAGTAGCTGG |
| IAA10R5 | GCAAGCTTTCAGGATCTGCCTCTTGTTG |
| IAA10R6 | GCGTCGACTCAGGATCTGCCTCTTGTTG |
| OsTIR1F1 | CAGAATTCATGGGGCGCGGCGGCTCGCGC |
| OsTIR1R1 | GCGGATCCCTACACAATCTGGACGCAGGCTG |
| OsTIR1L1F | GCGAATTCATGACGTACTTCCCGGAGGAG |
| OsTIR1L1R | GCGGATCCCTATAGGATTTTAACAAAATTTGG |
| OsTIR1L2F | GTGGATCCAAATGGTGTTCTTCCCGGAGGAG |
| OsTIR1L2R | GCGTCGACTTAGAACGTTGAGATGAATTCAG |
| OsTIR1L3F | GCCATATGATGAGCACTTCCCCCTCCTGCTC |
| OsTIR1L3R | GCGGATCCCTACAAGATGTTGACAAATGAAG |
| OsTIR1L4F | GCGAATTCATGTCCGAGGAGGACGACGACC |
| OsTIR1L4R | GCGGATCCTTATAGGATCTTCACGAATGGTG  AAGATCCTATAA |
| HAOsTIR1F1 | GCGGTACCGATGTACCCATACGATGTTCCAGATTACGCGATGGGGCGCGGCGGCTCGCGC |
| OsTIR1R2 | GCGTCGACCTACACAATCTGGACGCAGGCTG |
| OsTIR1F2 | CAGGATCCATGGGGCGCGGCGGCTCGCGC |
| OsIAA1F | GCGAATTCATGTCGGTGGAGACGGAG |
| OsIAA1R | GCGGATCCTCATTGAGCGGCTCTTG |
