# Supplementary material for: Rice Dwarf Virus P2 Protein Hijacks Auxin Signaling by Directly Targeting the Rice OsIAA10 Protein, Enhancing Viral Infection and Disease Development
Source: PLoS Pathog. 2016 Sep 8;12(9):e1005847. doi: 10.1371/journal.ppat.1005847 (PMC5015840; doi:10.1371/journal.ppat.1005847)
Supplement: S6 Table — (DOCX) [file ppat.1005847.s020.docx]

**S6 Table. Primers for Northern blot probes, q PCR, and RT-PCR.**

| **Primer name** | **Primer Sequence 5’-3’** | **Use** |
| --- | --- | --- |
| GUS-F8 | GATCTGACTAGTTTACGTCCTGT | RT-PCR |
| GUS-R811 | GTAGATATCACACTCTGTCTGGC |  |
| OsIAA10-F | ATGAGAGGAGGAGTAGCTGGGC |  |
| OsIAA10-R | TCAGGATCTGCCTCTTGTTG |  |
| q-OsIAA10-F | GGTTGCTGGATGGGTGAAGG | qPCR and Probe Synthesis |
| q-OsIAA10-R | CCTGTCCTCGTAGGTGAGCTGG |  |
| S2-F | AACTTTGCTTCGGTGGTTGCCCCTG |  |
| S2-R | GCTATACACATCATCGCCGTGGTGT |  |
| S11-F | ATGAGTGGAACATTACCCTTGG |  |
| S11-R | TTACTTACGCTTTGATTTGCG |  |
| q-OsIAA1-F | ACCAAGAGCCGCTCAATGAG | qPCR |
| q-OsIAA1-R | ATCACACGTGGGCGAACATC |  |
| q-OsIAA9-F | AAGAAAATGGCCAATGATGATCA |  |
| q-OsIAA9-R | CCCATCACCATCCTCGTAGGT |  |
| q-OsIAA11F | GCGCTGGTGAAGGTGAGCAT |  |
| q-OSIAA11R | ACGTACTCCAGGTCATCTCT |  |
| q-OSIAA21-F | GAAGGCACAGGTGGTAGGATG |  |
| q-OsIAA21-R | CCTGACTTCCCATTTGATTCACC |  |
| q-OsSAUR13-F | TTCGAGGTGCCATTGGTGTA |  |
| q-OsSAUR13-R | AGCCAAACTCCTCTTAGACATG |  |
| q-OsSAUR39-F | TACAGCTGATGGAGAGCGATT |  |
| q-OsSAUR39-R | TTTCCTCACCTGTGAATCCAA |  |
| q-OsSAUR44-F | ATGATCAGTGCCAAGAGACTCG |  |
| q-OsSAUR44-R | GGTGTACATGATGCAATGGCC |  |
| q-GH3.2-F | TCATGCCCGTCATGAACTTG |  |
| q-GH3.2-R | TCGTCTCCGACTTGATGAACAG |  |
| q-GH3.5-F | ATGACGATCTGCAGCTGTGAGGAGAC |  |
| q-GH3.5-R | GCACACACAATGGGATGCAGG |  |
| q-GH3.8-F | TTGGACCGTGTCCAAGAATCT |  |
| q-GH3.8-R | TCTTGCCACTAACTGACAGAGTTGA |  |
| q-OsEF1a-F | ACATTGCCGTCAAGTTTGCTG |  |
| q-OsEF1a-R | AACAGCCACCGTTTGCCTC |  |
| q-OsTIR1-F | ATTACATCCTCTCAGGCTGC |  |
| q-OsTIR1-R | ACCTTCCAGGATCGTTCATG |  |
| q-OsPIN1b-F | TGCACCCTAGCATTCTCAGCA |  |
| q-OsPIN1b-R | CCCTCCTCCCAAATTCTACTTC |  |
| q-OsPIN10a-F | CGGCTCTACCACAAGGGATTG |  |
| q-OsPIN10b-R | TCATAGTCCAAGAAGGATGTAGTACA |  |
| q-OsPR2-F | TTCAACGAGAACCAGAAG |  |
| q-OsPR2-R | TTAGAAATTGATGGAGTATGC |  |
| q-OsPR10-F | CCCTGCCGAATACGCCTAA |  |
| q-OsPR10-R | CTCAAACGCCACGAGAATTTG |  |
| q-OsWRKY13-F | TTTGGGAAAGCGTTGATTAGT |  |
| q-OsWRKY13-R | GCGCACACACACTCCAACTC |  |
| q-OsWRKY45-F | CGGGTAAAACGATCGAAAGA |  |
| q-OsWRKY45-R | TTTCGAAAGCGGAAGAACAG |  |
| q-OsJAZ12-F | ATCTGCCCGGTTTAGAGGAG |  |
| q-OsJAZ12-R | GGGCCAAAGAAATCTCAAAC |  |
